# Persulfated Ascorbic Acid Glycoside as a Safe and Stable Derivative of Ascorbic Acid for Skin Care Application

**DOI:** 10.3390/molecules29194604

**Published:** 2024-09-27

**Authors:** Ana Jesus, Marta Correia-da-Silva, Catarina Confraria, Sílvia Silva, Gonçalo Brites, Ana I. Sebastião, Mylène Carrascal, Madalena Pinto, Honorina Cidade, Paulo Costa, Maria T. Cruz, Emília Sousa, Isabel F. Almeida

**Affiliations:** 1 Associate Laboratory i4HB—Institute for Health and Bioeconomy, University of Porto, 4050-313 Porto, Portugal; anaaimjesus@gmail.com (A.J.); pccosta@ff.up.pt (P.C.); ifalmeida@ff.up.pt (I.F.A.); 2 UCIBIO—Applied Molecular Biosciences Unit, Laboratory of Pharmaceutical Technology, Faculty of Pharmacy, University of Porto, 4050-313 Porto, Portugal; 3 Laboratory of Organic and Pharmaceutical Chemistry, Department of Chemical Sciences, Faculty of Pharmacy, University of Porto, 4050-313 Porto, Portugal; m_correiadasilva@ff.up.pt (M.C.-d.-S.); catarinailc@gmail.com (C.C.); madalena@ff.up.pt (M.P.); hcidade@ff.up.pt (H.C.); 4 CIIMAR—Interdisciplinary Center of Marine and Environmental Research, Avenida General Norton de Matos S/N, 4450-208 Matosinhos, Portugal; 5 Laboratory of Pharmaceutical Technology, Department of Drug Sciences, Faculty of Pharmacy, University of Porto, 4050-313 Porto, Portugal; silvarfsilvia@gmail.com; 6 Faculty of Pharmacy, University of Coimbra, 3004-531 Coimbra, Portugal; g.sousabrites3@gmail.com (G.B.); anaisabelsebastiao@gmail.com (A.I.S.); 7 CNC—Center for Neurosciences and Cell Biology, 3004-504 Coimbra, Portugal; mylenecarrascal87@gmail.com; 8 Labor Qualitas, Tecnimede Group, Rua da Tapada Grande, Abrunheira, 2710-089 Sintra, Portugal; 9 UNIPRO—Oral Pathology and Rehabilitation Research Unit, University Institute of Health Sciences (CESPU), 4585-116 Gandra, Portugal

**Keywords:** ascorbic acid derivatives synthesis, safety profile, bioactivities, preformulation studies, cosmetics

## Abstract

The pursuit of cosmetic ingredients with proven efficacy and safety that meet consumer needs drives the advancement of new products. Ascorbic acid (AA) is utilized in cosmetic products, predominantly for its potent antioxidant properties. Nonetheless, its instability compromises its efficacy. In this work, ascorbyl 2-*O*-glucoside persulfate (AAGS) was synthesized, characterized, and evaluated regarding its safety profile and potential bioactivities and the results were compared to AA and its glycoside AAG. Pre-formulation studies were performed to assess the stability of the compounds and their compatibility with typical excipients commonly used in topical formulations. AAGS did not affect the metabolic activity of keratinocyte, macrophage, and monocyte cell lines, up to 500 µM. AAGS also exhibited a non-prooxidant and non-sensitizing profile and anti-allergic activity by impeding the allergen-induced maturation of THP-1 cells. When compared to AA and AAG, AAGS was shown to be more stable at pH values between 5 and 7, as well as superior thermostability and photostability. AAGS demonstrated higher stability in metal solutions of Fe(II) and Mg(II) than AA. AAGS demonstrated similar DPPH radical scavenging activity compared to AA. These results provide useful information for the development of new AA derivatives, highlighting AAGS as a novel cosmetic ingredient.

## 1. Introduction

Ascorbic acid (AA), usually known as vitamin C, is a water-soluble vitamin essential for human health [1,2] and is involved in many biological processes, promoting skin health [1,3,4]. In addition to its strong antioxidant properties [5], AA has already been reported for exhibiting photoprotective [6], wound healing [7], and depigmenting [8] properties. AA has also been studied for its ability to attenuate extrinsic aging, especially activated by UV radiation, and intrinsic aging of the skin. AA is effective in reducing oxidative stress through the neutralization of reactive oxygen species (ROS), due to the ability of ascorbate form to lose electrons, being converted into monodehydroascorbate, and subsequently into dehydroascorbate [5,9]. AA also promoted the expression and maturation of collagen-associated genes [6,10]. The formulation of cosmetic products containing AA presents some challenges, mainly due to its instability in aqueous solutions [11]. Other pitfall is the ability to exacerbate skin irritation reactions, mostly in sensitive skin [12]. The development of advanced formulations and the design of new AA derivatives have been pursued to improve parameters such as stability and cutaneous penetration, without compromising the bioactive properties [13,14]. Diverse AA derivatives have been proposed, such as, ascorbyl 2-*O*-glucoside (AAG), which duplicated collagen production (>12 µg/well) in human fibroblasts and demonstrated a better lightening effect on B16 cells at 4 mM when compared to 3-*O*-ethyl ascorbic acid, another AA derivative [15,16,17]. Sulfation, a molecular modification strategy applied in medicinal chemistry, helps to improve the solubility of the compounds in aqueous solutions and ameliorate the biological activities of the parent compound [18,19], which could help to overcome some drawbacks of AA. Indeed, sulfated bioactive compounds, such as sulfated polysaccharides [20] have already been reported for their antioxidant, anti-inflammatory, and anti-melanogenesis potential [18]. Building on our research group’s expertise in developing sulfated derivatives for topical use [21,22], we opted to persulfate the AAG derivative (AAGS) and explore its potential as a novel ingredient for skincare applications. Interestingly, our recent investigation into a persulfated resveratrol glycoside revealed that incorporating sulfate groups led to a safer compound with anti-allergic properties, inhibiting THP-1 cell maturation induced by the potent allergen 1-fluoro-2,4-dinitrobenzene (DNFB) [22]. In addition to AAGS, we also examined AAG and AA for comparative analysis to elucidate the impact of sulfate groups and the sugar moiety.

## 2. Results and Discussion

### 2.1. Chemical Synthesis

**Figure 1:**
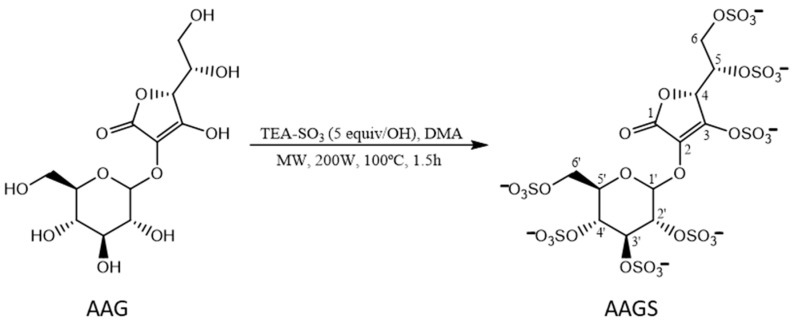
Synthesis of 2-*O*-α-*d*-glucopyranosyl-l-ascorbic acid persulfate (AAGS) from 2-*O*-α-*d*-glucopyranosyl-*l*-ascorbic acid (AAG). TEA-SO_3_: triethylamine-sulfur trioxide adduct, DMA: dimethylacetamide, MW: microwave, W: Watts, h: hours.

The synthesis of the new 2-*O*-α-d-glucopyranosyl-*l*-ascorbic acid persulfate (AAGS) was assisted by MW irradiation using the ascorbic acid derivative 2-*O*-α-*d*-glucopyranosyl-*l*-ascorbic acid (AAG) and triethylamine-sulfur trioxide (TEA-SO_3_) adduct (Figure 1). 

Initially, four equivalents/OH of TEA-SO_3_ were added, followed by a first cycle of 30 min of MW irradiation. Subsequently, it was confirmed by TLC that there was still raw Materials, so a second cycle of 30 min was carried out. Then, one more equiv/OH of TEA-SO_3_ was added and one more cycle of 30 min of MW irradiation was performed, finishing the reaction after 1.5 h, with only one product formed. Since DOWEX™ Cation Exchange Resin 50WX4 (Santa Cruz Biotechnology, Dallas, TX, USA, Na^+^ form) (50–100 mesh) was not successful in the conversion to sodium salts, the crude oil was dissolved in an aqueous solution of 30% NaOAc, resulting in the formation of a light-yellow solid, after insolubilization with ethanol. Subsequently, the product was purified through dialysis, yielding a light-yellow solid, 2-*O*-α-*d*-glucopyranosyl-l-ascorbic acid persulfate (AAGS). AAGS was obtained with a yield of 74% and a purity of 99.10% (HPLC-DAD) (Supplementary Materials, Figure S1). The structure of AAGS was established for the first time using infrared (IR) spectroscopy, nuclear magnetic resonance (^1^H and ^13^C NMR) and high-resolution mass spectrometry (HRMS). The IR spectrum of AAGS is presented in Supplementary Materials (Figure S2) and showed important bands at 1731 cm^−1^ (C=O), 1259 cm^−1^ (S=O), 1052 cm^−1^ (C-O-S), and 804 cm^−1^ (S-O), confirming the successful sulfation of AAG. The ^1^H and ^13^C NMR assignments of AAGS (Figures S3 and S4) were performed by comparison with the ^1^H and ^13^C NMR data of persulfated AA described in the literature [23] and are presented in Table S1 (Supplementary Materials). The ^1^H and ^13^C NMR assignments of the glucose residue were performed by comparison with the ^1^H and ^13^C NMR data previously reported for other sulfated glycosides [23]. Proton coupling (2.9 Hz) observed for the anomeric proton of the glucopyranosyl residue of AAGS demonstrated the expected linkage to the ascorbic acid aglycone. Finally, the presence of seven sulfate groups was unequivocal confirmed by HRMS (Figure S5).

### 2.2. Cell Culture Studies

#### 2.2.1. Cellular Metabolic Activity

**Figure 2:**
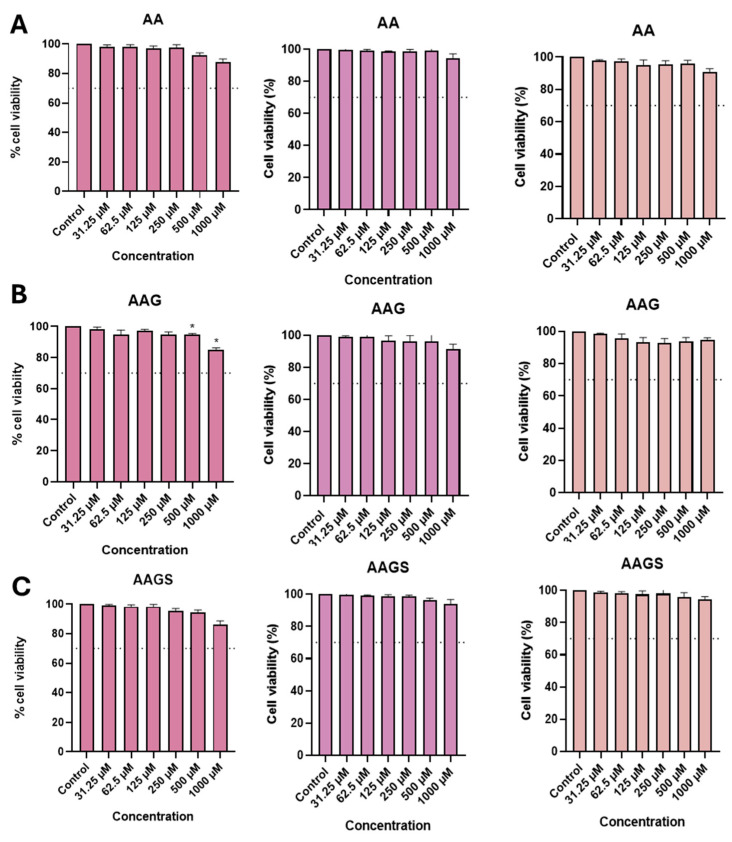
Cytotoxicity of compounds AA, AAG, and AAGS using a resazurin reduction assay in (**A**) HaCaT, (**B**) RAW264.7, and (**C**) THP-1 cells by comparison with the control. Bar graphs represent the mean ± SEM of at least three independent experiments performed in triplicate. Dashed line represents 70% of cell viability. Statistical analysis: one ANOVA with Dunnett’s multiple comparisons test where *p* < 0.05 was considered significant and * *p* < 0.05, relative to the control.

To study the effects of AA, AAG, and AAGS on cell viability, a resazurin assay was performed on three cell lines, representative of keratinocytes, macrophages, and dendritic cells (Figure 2A–C).

Concerning the RAW264.7 and THP-1 cell lines, all compounds proved to be safe at a maximum concentration of 1000 µM, with cell viability exceeding 90%. In the HaCaT cell line, AAG exhibited fluctuations in HaCaT metabolism at concentrations greater than or equal to 500 µM, although cell viability was maintained above 70%. Interestingly, the cytotoxic effects observed with AAG on the HaCaT cell line, as determined by the resazurin reduction assay, were effectively mitigated through its persulfation. Overall, the results suggest that AAGS did not adversely impact cell viability at the concentrations examined across the three cell lines studied, in accordance with the results also observed for other persulfates, such as the sulfated glycosylated resveratrol derivative previously described by us [21,22].

#### 2.2.2. Anti-Inflammatory Activity in RAW 264.7 Cell Line

**Figure 3:**
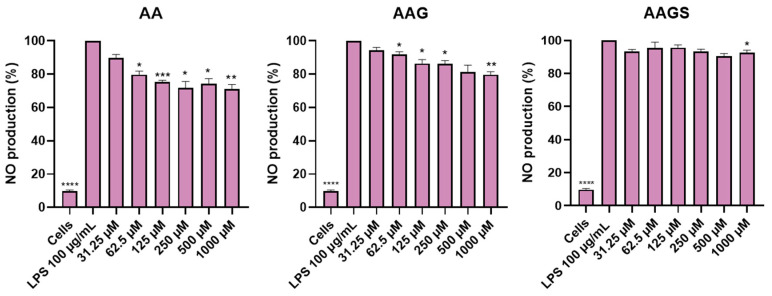
NO production in the presence of AA, AAG, and AAGS in macrophage cell line. Results are expressed as a percentage of NO production by cells treated with LPS. Each value represents the mean ± SEM of four independent experiments in duplicate. Statistical analysis: one ANOVA with Dunnett’s multiple comparisons test where *p* < 0.05 was considered significant and * *p* < 0.05, ** *p* < 0.01, *** *p*< 0.005, and **** *p*< 0.001, relative to the control.

The anti-inflammatory effect of the AA, AAG, and AAGS was assessed in lipopolysaccharides (LPS)-stimulated macrophages (Figure 3). While macrophages naturally generate baseline levels of nitrites, their production is enhanced when activated by LPS, a toll-like receptor 4 (TLR4) agonist, resulting in increased nitric oxide (NO) synthesis (LPS: 100%). The compounds did not show any pro-inflammatory activity. Interestingly, both AA and AAG inhibited NO production, with AA demonstrating the most promising NO inhibitory action, achieving a significant reduction of 30%. However, the results demonstrated that the presence of sulfate groups partially eliminated the anti-inflammatory activity towards NO production. 

#### 2.2.3. Inhibition of Maturation in THP-1 Cell Line

**Figure 4:**
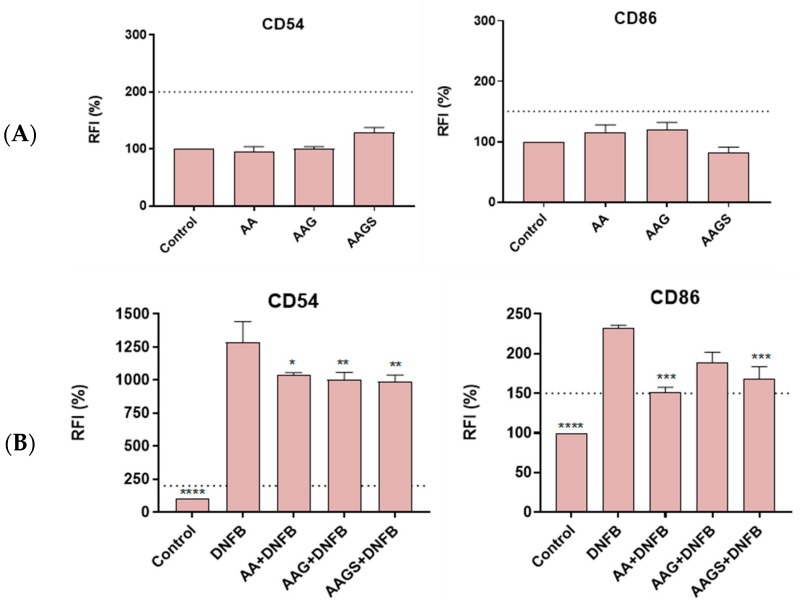
(**A**) Skin sensitization potential and (**B**) anti-allergic activity of AA, AAG, and AAGS toward the maturation of THP-1 cells induced by the allergen DNFB. The relative fluorescence intensity (RFI) of CD54 and CD86 expression was measured. Bar graph presents the mean ± SEM of at least three independent experiments. Dashed line indicates the established thresholds for CD54 (200%) and CD86 (150%) markers. The comparisons were performed using one-way ANOVA analysis with Dunnett’s multiple comparison post-test, where *p* < 0.05 was considered significant compared with the DNFB and * *p* < 0.05, ** *p* < 0.01, *** *p* < 0.005, and **** *p* < 0.001, compared to DNFB.

The necessity for alternatives to animal testing has been acknowledged for assessing the hazard of chemicals to human health, particularly within the cosmetic industry, owing to the full enforcement of testing and marketing bans on animal experimentation under the European Cosmetics Regulation. Adhering to OECD guidelines for skin sensitization evaluation, the potential of AA, AAG, and AAGS to induce skin sensitization was assessed by monitoring the upregulation of co-stimulatory molecules CD54 and CD86 in THP-1 cells (Figure 4A,B). All compounds exhibited relative fluorescence intensity (RFI) values below the thresholds (CD54: 200% and CD86: 150%) outlined by the OECD guideline In Vitro Skin Sensitisation: Human Cell Line Activation Test (*h*-CLAT) [24], confirming the absence of skin sensitization potential for all compounds. 

Moreover, the potential of these compounds to mitigate skin sensitization was explored using DNFB, a strong allergen (Figure 4B). All the compounds inhibited the DNFB-induced maturation profile of THP-1 cells. Interestingly, the persulfation of AAG was found to be beneficial for a better inhibitory response of the allergen-induced cell maturation, with RFI for AAGS of 986.89% ± 80.51%, and 168.90% ± 16.30%, in contrast to the RFI for AAG of 995.78% ± 80.51%, and 189.198% ± 17.43%, both for the expression of CD54 and CD86 membrane cell markers, respectively. Overall, AA and derivatives could be considered as non-sensitizer molecules. Considering the ability to significantly revert the CD54 increase induced by DNFB on THP-1 cells, the three ascorbic derivatives were found to exhibit anti-allergic potential. Regarding CD86 cells, AA and AAGS also exhibited anti-allergic profile, in contrast to AAG. These findings suggest that the sulfated derivative of AAG could be used to prevent or minimize the sensitization potential of allergens, thereby reducing the harmful effects that may result from the contact between the skin and allergens.

#### 2.2.4. Mitochondrial Antioxidant Activity

**Figure 5:**
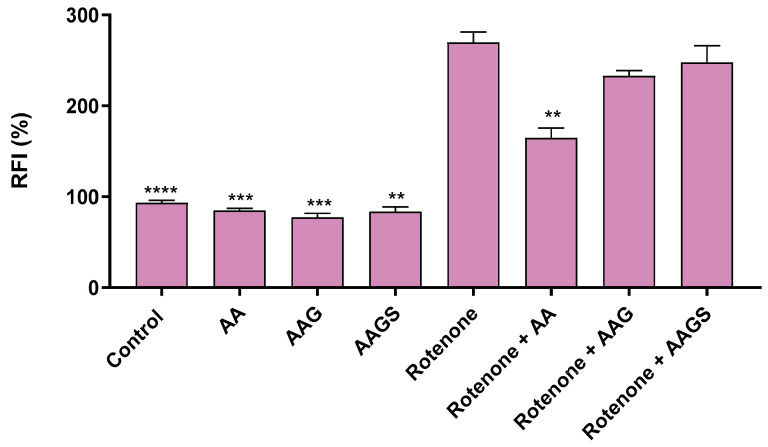
Mitochondrial antioxidant activity of AA, AAG, and AAGS (500 μM) towards the rotenone (20 μM)-induced oxidative stress in RAW264.7 cell. The relative fluorescence intensity (RFI) of the fluorescent probe was measured. Bar graph presents the mean ± SEM of at least independent experiments. Statistical analysis: one ANOVA with Dunnett’s multiple comparisons test where *p* < 0.05 was considered significant and ** *p* < 0.01, *** *p* < 0.005, and **** *p* < 0.0001, compared to rotenone.

Mitochondria are a significant source of ROS, such as superoxide anion (O_2_^−^), and once that oxidative stress has been related to a variety of skin diseases [25], flow cytometry methodology was selected to measure mitochondrial O_2_^−^ anion in live cells in the presence of the AA’s family of compounds. Considering this, the mitochondrial antioxidant activity was also accessed in rotenone-stimulated macrophage cells (Figure 5).

At a concentration of 500 μM, both AA and its derivatives exhibited a safe and inert profile. No pro-oxidant response was detected, as evidenced by the absence of an increase in fluorescence, which was intrinsically correlated with superoxide anion (O_2_^−^) production. Rotenone at a concentration of 20 μM acts as an oxidative stress inducer by inhibiting complex I in the mitochondria [26]. As expected, this inhibition resulted in a threefold increase in the detected fluorescence, with a relative fluorescence intensity (RFI) of 269.70 ± 11.63%. These results are consistent with AA’s well-established antioxidant activity of AA [27]. These findings corroborate previous literature describing the diverse beneficial effects of AA on the mitochondria, highlighting its efficacy in combating mitochondrial ROS [26]. Derivatives AAG and AAGS did not demonstrate an ability to exert antioxidant activity in mitochondria, suggesting that the 3-OH and the 2-OH groups present in AA are important for the antioxidant activity [28,29]. Despite having no mitochondrial antioxidant activity, AAG and AAGS showed no pro-oxidant profile at the concentration tested.

#### 2.2.5. DPPH Assay

The ability of the compounds to scavenge DPPH radicals was evaluated (Table S2). The results showed that all three compounds reduced DPPH at the tested concentrations, with AA being the most potent DPPH radical scavenger. Therefore, despite the lack of mitochondrial antioxidant activity, AAG and AAGS exhibited the ability to scavenge DPPH radicals. Interestingly, although a decrease of activity was observed when comparing AAGS (EC_50_ = 5.28 ± 0.36 µM) with AA (EC_50_ = 1.54 ± 0.21 µM), an enhancement of the DPPH scavenging activity was observed when comparing AAGS with AAG (EC_50_ = 9.15 ± 0.13 µM), suggesting that the presence of sulfate groups could be beneficial for the antioxidant activity. 

### 2.3. Preformulation Studies 

#### 2.3.1. Stability Studies

##### pH

**Figure 6:**
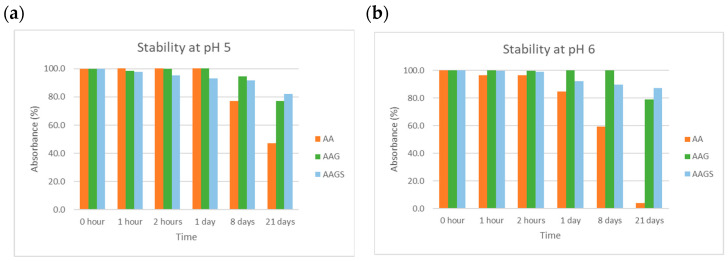
Stability studies at different pH values for compounds AA, AAG, and AAGS in aqueous solutions at (**a**) pH 5 and (**b**) pH 6. The results are expressed as the percentage of absorbance normalized to the absorbance at time t = 0, in triplicate.

The stability of the compounds was examined at pH values ranging from 2 to 8. Figure 6 represents the results obtained at pH 5 and 6, which are typical pH values of skin care formulations and are close to skin pH. The remaining results are presented in the Supplementary Materials (Figure S6). 

AA was unstable at acidic and neutral pH (Figure 6) with variations in absorbance > 90% (Figure S5). AA degradation occurs quickly, and AA degrades irreversibly into a biologically inactive form (2,3-diketo-l-gulonic acid) [27]. AAG was more stable than AA at pH ≥ 5 and behaved similarly to AAGS. Overall, AAGS demonstrated improved stability covering a larger range of pH values (pH = 2–6), when compared to AA and AAG. 

##### Temperature

**Figure 7:**
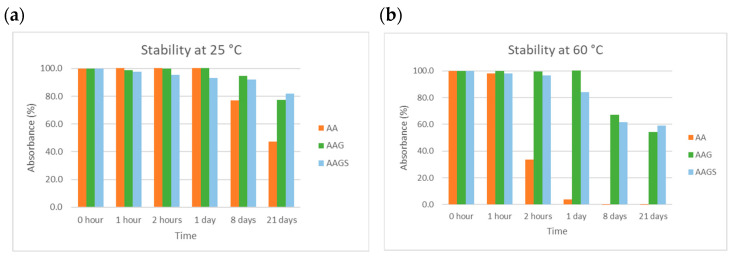
Stability studies for AA, AAG, and AAGS in aqueous solution at pH 5 at different temperatures: (**a**) 25 °C and (**b**) 60 °C. Results are expressed as % of absorbance normalized to the absorbance at the time t = 0, in triplicate.

The compounds were examined regarding their stability at different temperatures, in aqueous solution at pH 5. Figure 7 presents the results obtained for the three compounds at 25 °C and 60 °C. The results for the remaining temperatures (4 and 40 °C) are shown in the Supplementary Materials (Figure S7).

The results showed that AA decomposed with time at all the temperatures tested. Storage at 25 °C was the only condition that minimized AA degradation (absorbance < 50%) whereas storage at lower (4 °C) and higher (40 °C and 60 °C) temperatures encouraged a strong degradation with time (e.g., absorbance < 30% in 2 h at 60 °C). Compared to AA, both AAG and AAGS derivatives demonstrated greater stability across all tested temperature ranges. The newly synthesized AAGS derivative once again showed the highest level of stability among the compounds.

##### Metals

**Figure 8:**
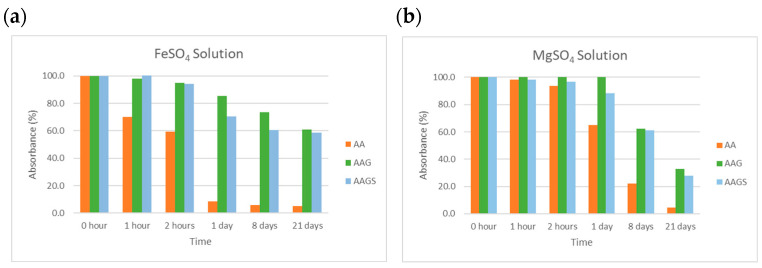
Stability studies for compounds AA, AAG, and AAGS at pH 5, for different metals, (**a**) Fe(II), and (**b**) Mg(II). Results are expressed as % of absorbance normalized to the absorbance at the time t = 0, in triplicate.

Solutions of AA derivatives were prepared with different metal solutions of Ca(II), Cu(II), Fe(II), Fe(III), and Mg(II), and analyzed over time. Figure 8 shows the results obtained for Fe(II) and Mg(II). The remaining studies of the metal solutions of Fe(III), Cu(II), and Ca(II) are presented in the Supplementary Materials (Figure S8).

**Figure 9:**
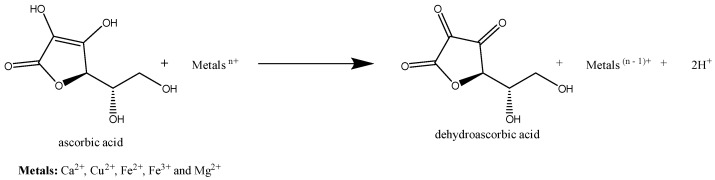
Oxidation reaction of ascorbic acid (AA) in presence of metals.

The levels of AA stability in the presence of all metal solutions were lower than those for AAG and AAGS, with a decrease in AA stability over time. This instability was more evident for Cu(II), Fe(II), and Fe(III), with a loss of absorbance > 80%. In the presence of Mg(II) and Ca(II), the AA concentration did not suffer a substantial decrease in the first 2 h; however, its instability was well noted after 1 d. In the presence of catalytic amounts of metal ions, AA is rapidly oxidised to dehydroascorbate [30,31] (Figure 9). AAG showed higher stability against Fe(III) and Cu(II) degradation than AA, remaining reasonably stable (loss of absorbance < 30%) at the end of 1 d. The results indicate that the glycosyl sugar moieties attached to AA impeded the metal-catalysed oxidation process. Specifically, the introduction of a glycosyl group at the C-2 position of AA created a steric hindrance, limiting the ability of metal ions to approach or bind to AA. AAGS followed the AAG behavior in the first few hours of analysis for Mg(II) and Fe(II), but in the presence of Ca(II), Cu(II), and Fe(III), AAGS suffered an accentuated decrease in absorbance after one day (loss of absorbance > 50%). 

##### Photostability

**Figure 10:**
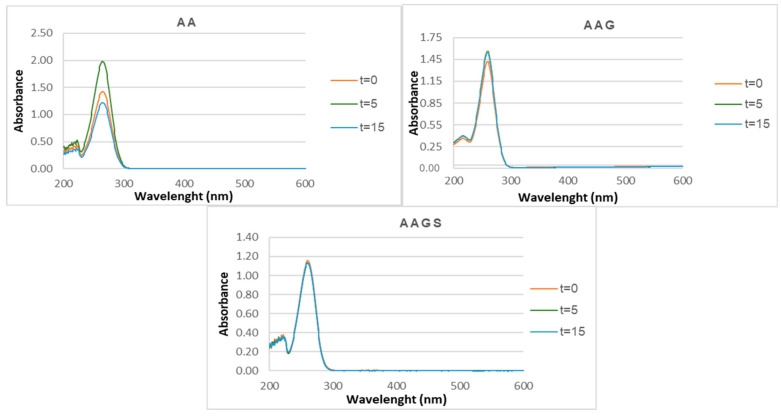
Photostability studies for compounds AA, AAG, and AAGS in aqueous solution, after irradiation up to 15 min with a lamp as light source with an irradiance of 0.62 mW/cm^2^ and 0.74 mW/cm^2^ of UVA and UVB light, respectively.

Compounds AA, AAG, and AAGS were exposed to UVA + UVB radiation to evaluate their photostability. Figure 10 shows the absorbance spectra of each compound before and after irradiation.

Initially, AA exhibited a hyperchromic effect, with its absorbance increasing by nearly 50% after 5 min of UV exposure. Subsequently, a hypochromic effect was observed following 15 min of UV radiation exposure. These results are in accordance with recently reported results [32]. For AAG, a slight variation in the absorbance was observed, whereas for AAGS, the variations in absorbance values were practically imperceptible, demonstrating a better photostable profile than AA and AAG. 

##### Compatibility with Excipients

Table 1 shows the study of compatibility between AA, AAG, and AAGS and some excipients from different categories, which are typically used in topical formulations obtained through the analysis of the DSC curves. The thermograms of the compounds and mixtures are in the Supplementary Materials (Figure S9a–x).

In the thermograms of AA and AAG, one endothermic peak is observed, which can be attributed to the melting of the tested compounds, as expected according to the literature [31,33]. The thermogram of AAGS is characterized by two minor endothermic peaks and a pronounced exothermic peak, all of which are probably related to alterations in the chemical structure of AAGS, possible cleavage of bonds, and melting of the compound. Initially, the AA, AAG, and AAGS thermal behavior in the calorimetric curve demonstrated a well-defined peak at 197.1 °C, 176.4 °C, and 200.5 °C, respectively. After analyzing the thermograms from the 1:1 weight/weight (*w*/*w*) mixtures, the three AA derivatives showed minimal or no interaction with five excipients, while demonstrating moderate to strong interference with two excipients. Among these combinations, the more compatible were EDTA with AAG, hydroethylcellulose (HEC) with AAGS, miglyol 812 (MIG) with AAG and AAGS, and tocopherol (TP) with AAG and AAGS, since no relevant changes were observed. Potassium cetyl phosphate (PCP) was demonstrated to be strongly incompatible with all AA derivatives. Weak interferences were seen for stearyl alcohol (SA) with AAG and AAGS, Carbopol 980 (CP) with AA and AAGS, EDTA with AA and HEC with AAG. MIG did not alter any T_peaks_ of AA and derivatives. Tocopherol is an antioxidant and has been shown to be compatible with AA and its derivatives. It has been reported that the quantity of material used, especially in active-excipient mixtures, affects the peak shape and enthalpy [29,34], which can be attributed to the mixtures, which lowers the purity of each component and may not necessarily indicate potential incompatibility. Based on the comprehensive results summarized in Table 2, several beneficial conclusions can be drawn regarding the new AA derivative, AAGS, in comparison to its parent compound. AAGS exhibited reduced cytotoxicity in keratinocyte cells and demonstrated enhanced stability across a broad pH range, at various temperatures, and when subjected to UV radiation.

## 3. Materials and Methods

### 3.1. Materials

Ascorbic acid, ascorbic acid 2-glucoside, tocopherol, iron (III) chloride, copper (II) sulfate pentahydrate, magnesium sulfate monohydrate, ethylenediaminetetraacetic acid (EDTA), citric acid, rutin, 1,1-diphenyl-2-icryl-hydrazyl (DPPH), allergen 1-fluoro-2,4-dinitrobenzene (DNFB), resazurin sodium salt, tetrabutylammonium bromide (TBAB), and the sulfur trioxide triethylamine complex (TEA-SO_3_) were obtained from Sigma-Aldrich (St. Louis, MO, USA). Miglyol 812 and stearyl acid were obtained from Acofarm (Padrid, Spain), carbopol 980 from Noveon (San Marcos, TX, USA), and hydroxyethyl cellulose form Fragon (Rotterdam, The Netherlands). Ascorbic acid 2-glucoside was obtained from DKSH (Zurich, Switzerland). Potassium chloride, trisodium citrate dihydrate, and iron (II) sulfate heptahydrate were obtained from Merck (Darmstadt, Germany), and hydrochloric acid was obtained from Fischer Chemicals (Waltham, MA, USA). Sodium hydroxide and disodium hydrogen phosphate were obtained from VWR Chemicals (Leicestershire, UK), phosphoric acid from Panreac (Barcelona, Spain), calcium hydroxide from Cromoline (Diadema, Brazil), and potassium cetyl phosphate from AAKO (Leusden, The Netherlands). The solvents used were of product pro analysis or HPLC grade (Sigma-Aldrich and Fluka, St. Louis, MO, USA,). The antibodies used for staining were anti-CD86 (clone IT2.2, reference 305414) and anti-CD54 (clone HA58, reference 353111), which were both purchased from BioLegend (San Diego, CA, USA). MitoSOX Red Mitochondrial Superoxide Indicator (MitoSOX) was purchased from Invitrogen (Carlsbad, CA, USA). 

The microwave (MW) reaction was performed in open reaction vessels (20 mL) in a MicroSYNTH 1600 synthesizer from Milestone (Thermo Unicam, Lisboa, Portugal). The internal reaction temperature was controlled using a fiber-optic probe sensor. The reactions were monitored by thin-layer chromatography (TLC) using Merck silica gel 60 (GF254) plates. The solvents were evaporated using a rotary evaporator under reduced pressure (Buchi Waterchath B-480, Buchi, New Castle, DE, USA). Purification of the synthesized compound was carried out either by DOWEX™ Cation Exchange Resin 50WX4 (Na^+^ form) (50–100 Mesh) and/or dialysis Spectra/Por 6 regenerated cellulose MWCO 1000 membranes. The peak purity was determined by HPLC analysis. The melting point was determined using a Fisatom 430 melting point apparatus and data are uncorrected. IR spectra were measured on an ATI Mattson Genesis series FTIR (software: WinFirst v.2.10) spectrophotometer in KBr microplates (cm^−1^). ^1^H and ^13^C NMR spectra were performed in the Department of Chemistry of the University of Aveiro and were taken in DMSO-*d*_6_ at room temperature, on Bruker Avance 300 instruments (Billerica, MA, USA) 300.13 for ^1^H and 75.47 MHz for ^13^C). Chemical shifts were expressed as δ (ppm) values relative to tetramethylsilane (TMS) as an internal reference. The coupling constants (J) are reported in hertz (Hz). The assignment abbreviations are as follows: singlet (s), doublet (d), triplet (t), quartet (q), multiplet (m), doublet of doublets (dd), broad doublet (brd), and broad triplet (brt). Analyses were performed on an Orbitrap Exploris 120 mass spectrometer (Thermo Fischer Scientific, Bremen, Germany) controlled by Orbitrap Exploris Tune Application 2.0.185.35 and Xcalibur 4.4.16.14. The capillary voltage of the electrospray ionization source (ESI) was set to 3.4 kV and 2 kV for positive and negative mode. The capillary temperature was 320 °C. The sheath and auxiliary gas flow rates were at 5 (arbitrary unit as provided by the software settings). The resolution of the MS scan was 60,000. Data-dependent MS/MS was performed on HCD using nitrogen as gas with collision energy settings of 30 V. The m/z range values were 200–2000 Da. The resolution of SIM MS scan was 60,000. MS data handling software (Xcalibur QualBrowser software Thermo Fischer Scientific) was used to search for predicted metabolites by their m/z value and MS/MS value. HPLC-DAD analyses were performed on Thermo Scientific SpectraSYSTEM equipped with a SpectraSYSTEM UV-8000 DAD detector, a SpectraSYSTEM P4000 pump, and a SpectraSYSTEM AS3000 autosampler, and the used software was ChromQuest 5.0™. The analyses were conducted on a Fortis BIOC18 column ((Fortis BIOC18, Fortis Technologies, Pasig, Philippines) 5 µm Fortis BIO C18, 250 × 4.6 mm). HPLC ultrapure water was generated by a Milli-Q purification system (Millipore, Burlington, MA, USA). The mobile phase used was 25 mM of tetrabutylammonium (TBAB) and acetonitrile (38:62) and was filtered through a 0.45 µm filter and degassed for 15 min in an ultrasonic bath before use (Sonorex Digitec, Bandelin, Berlin, Germany), with a constant flow rate of 1.0 mL/min and the injection volume was 20 µL. A Jasco V-650 UV–visible spectrophotometer dual beam (Jasco, Tokyo, Japan) was used, equipped with a deuterium UV lamp and Vis QI interfaced with the Windows program of a computer via Spectra Manager^TM^ II software (https://jascoinc.com/products/spectroscopy/molecular-spectroscopy-software/ (accessed on 22 September 2024)) data system program. Two open-top UV quartz cells with a length of 1 cm and wavelength of 280 nm were used. An ultrasonic device (ultrasonicator) was used to dissolve the antioxidants in the solution samples (Selecta, Abrera, Spain). A pH meter was used to measure the pH of each buffer solution (Jenway 3510, Chelmsford, UK). The light source was a fluorescent lamp (Osram, Munich, Germany, Ultra-Vitalux 300 W) that was designed to produce artificial daylight combining vis and UV outputs and a radiometer (Arimed, Cosmedico–Medical Systems, Physikalisch UVM-7, Armagh, UK). 

### 3.2. Synthesis

#### Synthesis of 2-O-α-d-glucopyranosyl-l-ascorbic Acid Persulfate

Ascorbic acid 2-glucoside (AAG) (1.0 g; 2.96 mmol) was dissolved in dimethylacetamide (22 mL) and triethylamine-sulfur trioxide adduct (18.8 g; 103.46 mmol; 5 equiv/OH) was added. The mixture was heated at 100 °C for three cycles of 30 min under MW (200 W). After cooling, triethylamine was added until pH 8 (10 mL) was reached, and the obtained solution was poured into acetone (80 mL) and left at 4 °C overnight. The crude oil formed was washed with acetone and ether, dissolved in a minimum volume of water, and applied to a DOWEX™ Cation Exchange Resin 50WX4 (Na^+^ form). After evaporation, the oil was obtained and dissolved in an aqueous solution of 30% NaOAc (5 mL). A light-yellow solid was insolubilized with ethanol and further purified by dialysis using a Spectra/Por 6 regenerated cellulose MWCO 1000 membrane to obtain a light-yellow solid corresponding to 2-*O*-α-d-glucopyranosyl-l-ascorbic acid persulfate (AAGS). 

**2-*O*-α**-*d*-**glucopyranosyl**-*l*-**ascorbic acid persulfate (AAGS):** Yield: 74%; mp 139–142 °C (water); IR (KBr) ʋmax: 1731, 1259, 1052, 804 cm^−1^; ^1^H NMR (DMSO-*d*_6_, 300.13 MHz) *δ*: 5.53 (1H, *d*, *J* = 2.9 Hz, H-1′), 4.62–4.52 (2H, *m*, H-2′, H-3′), 4.31 (1H, *brt*, H-4′), 4.18–4.13 (1H, *m*, H-5′), 4.10–3.94 (4H, *m*, H-4, H-5, H-6a, H-6′a), 3.83–3.70 (2H, *m*, H-6b, H-6′b). ^13^C NMR (*DMSO-d*_6_, 75.47 MHz) *δ*: 176.5 (C-1), 173.6 (C-3), 114.2 (C-2), 97.2 (C-1’), 79.2 (C-5′), 75.4 (C-4), 74.2 (C-3′), 74.0 (C-4’), 71.0 (C-2’), 70.4 (C-5), 65.9 (C-6), 63.3 (C-6′). HRMS (ESI+) m/z: [M+2Na+K] calcd for C_12_H_11_Na_9_O_32_S_7_K 1136.598923, found 1136.59462.

### 3.3. Cell Culture

The human keratinocytes (HaCaT, CLS 300493, Eppelheim, Germany) cell line was cultured with Dulbecco’s Modified Eagle’s Medium (DMEM) (Sigma-Aldrich, St. Louis, MO, USA), supplemented with 10% (*v*/*v*) of heat-inactivated fetal bovine serum (FBS), 1% (*v*/*v*) antibiotic solution (from a 10,000 U/mL penicillin and 10,000 μg/mL streptomycin stock) (Gibco, Carlsbad, CA, USA), 3.7 g/L sodium bicarbonate, and 1 mM sodium pyruvate (Sigma-Aldrich, St. Louis, MO, USA). The cell culture of keratinocytes was detached with EDTA solution 1X (Sigma-Aldrich, St. Louis, MO, USA) when the cells reached 70–80% confluence in cell culture flasks. The mouse macrophage cell line (RAW 264.7, ATCC TIB-71, Manassas, VA, USA) was cultured in DMEM supplemented with 10% (*v*/*v*) non-inactivated FBS, 1% (*v*/*v*) antibiotic solution, 1.5 g/L sodium bicarbonate, and 1 mM sodium pyruvate. Macrophages were mechanically detached with a cell scraper and subcultured in (1:10) proportion of cellular suspension to a final volume of DMEM. The human monocytic cell line THP-1 (ATCC^®^ TIB-202™, American Type Culture Collection, Manassas, VA, USA) was cultivated in RPMI 1640 supplemented with 10% inactivated FBS, 25 mM (d)-glucose, 10 mM HEPES, 1 mM sodium pyruvate, 100 U/mL penicillin, and 100 μg/mL streptomycin, and maintained at a cell density of 0.4 × 10^6^ cells/mL. Cells were subcultured every 2 or 3 days and maintained in culture for 2–3 months, according to ATCC^®^ instructions.

#### 3.3.1. Cellular Metabolic Activity in Different Cell Lines

The metabolic activity of human epidermal keratinocytes (HaCaT), murine macrophages (RAW264.7), and human monocytes (THP-1) was assessed using a resazurin reduction assay [35,36]. Cells were plated at a density of 1.0 × 10^5^ cells/mL (HaCaT and THP-1), and 5.0 × 10^4^ cells/mL (RAW264.7) in a 96-well plate and allowed to stabilize overnight. The cells were then exposed to different concentrations of AA, AAG, and AAGS and incubated at 37 °C for 24 h. After 20 h, resazurin solution (50 μM) was added and further incubated for 4 h. The absorbance was read at 570 and 620 nm using a SpectraMax Plus 384 absorbance microplate spectrophotometer (Molecular Devices, San Jose, CA, USA). All experiments were performed in triplicate, with a minimum of three independent assays, and were graphically represented as the percentage of cell viability versus the concentration of the chemicals compared with the control.

#### 3.3.2. Anti-Inflammatory Activity in Macrophage Cell Line

Nitric oxide (NO) production was measured by quantifying the accumulation of nitrites in culture supernatants using the Griess assay [37,38]. Cells were plated at a density of 5.0 × 10^4^ cells/mL in a 96-well plate and allowed to stabilize overnight. The cells were exposed to different concentrations of AA, AAG, and AAGS and pre-incubated for 1 h. Then, a solution of LPS (100 ng/mL) was added, followed by an incubation period of 24 h. A volume of 150 μL of cell culture supernatant was collected in a new 96-well plate, and 150 μL of Griess reagent ([1:1] of 1% (*w*/*v*) sulfanilamide in 5% (*v*/*v*) phosphoric acid and 0.1% (*w*/*v*) naphthylethylenediaminedihydrochloride]) was added. After a 30 min incubation period at room temperature, protected from light, the absorbance was read at 550 nm using a microplate spectrophotometer. The concentration of nitrites present in the supernatants was determined by interpolation of the absorbance of each sample in a sodium nitrite standard curve. The results of four independent experiments, in duplicate, were expressed as the percentage of NO production by cells versus the concentration of the chemicals compared with LPS (100%). 

#### 3.3.3. Inhibition of THP-1 Maturation Profile Induced by the Strong Allergen DNFB

Following OECD guidelines, researchers utilized the THP-1 human monocyte cell line as a substitute for dendritic cells (DCs) to assess skin sensitization potential [24]. Adapting OECD Test Guideline No. 442E [24], cell density was adjusted to 5.0 × 10^5^ cells/mL and allowed to stabilize overnight at 37 °C. Then, 1.5 mL/well of cell suspension was added to a 12-well plate, and solutions of AA, AAG, and AAGS (500 µM) were applied. After 1 h of pre-incubation, a solution of DNFB was added (8 µM), and the cells were further incubated for 24 h. Afterwards, the cells were centrifuged at 300× *g* for 5 min, the supernatant was discarded, and the cell pellet was washed with PBS containing 1% FBS (2 × 1 mL) and centrifuged. The cell pellet was resuspended in 1 mL of PBS/1% FBS and 200 µL were collected in two microtubes: 100 µL for the unstained (UNST) and 100 µL for the stained (CD5486) conditions. For each stained condition, anti-CD54-APC and anti-CD86-Alexa Fluor 488 (BioLegend, London, UK) were added, and the cells were incubated for 30 min at 4 °C, protected from light. Then, cells were washed twice with 1 mL PBS/1% FBS solution and centrifuged. The cell pellet was further resuspended in 100 µL of PBS and analyzed by flow cytometry using a BD Accuri™ C6 cytometer (San Jose, CA, USA). 

#### 3.3.4. Mitochondrial Antioxidant Activity

Mitochondrial superoxide (O_2_^−^) generation was determined using the MitoSOX Red kit, according to the manufacturer’s instructions. Briefly, 5.0 × 10^5^ cells/mL were plated in a 12-well plate and incubated for 24 h at 37 °C. The cells were then exposed to the AA, AAG and AAGS solution (500 µM), and after 1 h, rotenone (20 μM) was added, followed by incubation for an additional 5 h. The cells were then carefully detached, transferred to microtubes, and centrifuged at 500× *g* for 3 min. The cell pellet was washed with HBSS solution (2 × 1 mL), resuspended in PBS solution, and 300 µL was collected in two microtubes for unstained (150 µL) and stained (150 µL) conditions. The stained cells were incubated with 100 μL of freshly prepared MitoSOX solution 2.5 µM at 37 °C for 15 min in the dark. The stained cells were washed with HBSS solution (2 × 1 mL), and the cell pellet was resuspended in 100 μL of PBS and analyzed by flow cytometry using a BD Accuri™ C6 cytometer (San Jose, CA, USA).

#### 3.3.5. Flow Cytometry Analysis 

CD86 and CD54 levels were analyzed by flow cytometry using the acquisition channels FL1 and FL4. Mitochondrial O_2_^−^ production was measured by flow cytometry using the acquisition channel FL2. The relative fluorescence intensity (RFI) of CD86 and CD54 (THP-1 maturation assay) and MitoSOX (mitochondrial antioxidant activity) in the control and chemical-treated cells, based on the geometric mean fluorescence intensity (*MFI*), was calculated according to the following equation:

$$RFI=\frac{MFI\;of\;chemical\;treated\;cells-MFI\;of\;chemical\;treated\;unstained\;cells}{MFI\;of\;control\;treated\;cells-MFI\;of\;control\;unstained\;cells\;}\times 100$$

The results of at least three independent experiments were represented as a percentage of the control RFI, which was assumed to be 100%. For THP-1 maturation assay, the samples were designated as skin sensitizers if the RFI (%) of CD54 and CD86 was equal or superior to 200% and 150%, respectively. 

### 3.4. DPPH Activity 

Stock solutions of each compound (100 µM) and DPPH solution (150 µM) were prepared in ethanol. Different concentrations of antioxidant solutions (0.1–50 µM) were prepared to test their antioxidant effect. In a 96-well plate, 100 µL of DPPH working solution was added to a 100 µL sample solution and incubated at room temperature for 15 min in the dark. The absorbance of each sample was measured at 517 nm, in a microplate reader. For the negative control, 100 μL ethanol was added instead of the antioxidant solution. Rutin was used as the positive control. Inhibition curves were constructed, and the half maximal effective concentration (EC_50_) values were calculated for all samples by linear regression for the three independent assays performed. 

### 3.5. Preformulation Studies

#### 3.5.1. Stability

##### pH

Buffer solutions with pH values between 2 and 8 were prepared and their pH was measured. The buffer solutions were prepared according to Portuguese Pharmacopeia 11.0 specifications. Each compound solution (100 µM) buffered at a specific pH was prepared in triplicate. The solutions were stored at room temperature and protected from light. Aliquots of 3.00 mL were transferred to quartz cuvettes at specific time points, and their absorbance was measured by ultraviolet/visible (UV–vis) spectrophotometry. The blank for each pH corresponded to 3.00 mL of the respective buffer.

##### Temperature

Each compound solution (100 µM) buffered at pH 5 was prepared in triplicate. The other three samples were stored at 4 °C, 25 °C, 40 °C, and 60 °C, protected from light. Aliquots of 3.00 mL were transferred to quartz cuvettes at specific time points, and their absorbance was measured by UV–vis spectrophotometry. The blank of each pH corresponded to 3.00 mL of respective buffer. Stability was determined in terms of the change in the maximum peak absorbance.

##### Metals

Five independent metal solutions (100 µM) were prepared in a buffer at pH 5, in triplicate. To the compound solutions, a volume of metal solutions was added to a final proportion of 1:1. The stability in the presence of different metals was analyzed at specific time points. The solutions were stored at room temperature in the dark. Aliquots of 3.00 mL were transferred to quartz cuvettes at specific time points to measure their absorbance using UV–vis spectrophotometry. Solutions of each metal were used as blanks.

##### Photostability

Three independent compound solutions (100 µM) were prepared and exposed to light. A volume of 50 mL of each sample replicate was exposed side-by-side under a UV lamp at a 42 cm distance through an appropriate duration of time. These conditions were monitored using calibrated radiometers. The lamp used as light source for the photostability assay emitted an irradiance of 0.62 mW/cm^2^ and 0.74 mW/cm^2^ of UVA and UVB light, respectively. A UV lamp (Osram, Ultra-Vitalux, 300 W) was positioned at a height of 54 cm. The UV lamp was turned on 1 h before the experiment, and the UVA and UVB irradiances were measured, maintaining the controlled temperature. At the end of the exposure period, the absorbance spectrum was measured between 200 nm and 800 nm using a UV–vis spectrophotometer. The exposed samples were analyzed concomitantly with protected samples (dark controls). Photostability was evaluated by comparing the UV–vis spectra at each evaluation time for each antioxidant.

##### Compatibility with Excipients

A differential scanning calorimeter (DSC) 200F3 Maia (Netzsh-Gerätebau GmbH, Selb, Germany) was used for thermal analysis of the ingredients and mixtures. Typical excipients of topical formulations with different functions were selected for this study. The physical mixtures between compounds and the excipients were prepared in a ratio of 1:1 (*w/w*). Individual samples and the physical mixtures were weighed directly in the pierced DSC aluminum pan and scanned in the temperature range of 20–400 °C under nitrogen atmosphere with flow of 40 mL/min, with a heating rate of 10 °C/min. An empty aluminum pan was used as reference. The onset temperature and melting enthalpy (ΔH) were calculated using Proteus Analysis software (Version 6.1, NETZSCH-Gerätebau GmbH, Germany). The DSC cell was calibrated (sensitivity and temperature calibration) with Hg (m.p. −38.8 °C), In (m.p. 156.6 °C), Sn (m.p. 231.9 °C), Bi (m.p. 271.4 °C), Zn (m.p. 419.5 °C), and CsCl (m.p. 476.0 °C) as standards. 

### 3.6. Statistical Analysis

Statistical analyses were performed using GraphPad Prism8 for Windows (GraphPad Software, San Diego, CA, USA; www.graphpad.com). The results were expressed as mean ± standard error of the mean (SEM) of at least three independent experiments. For the obtained results, the comparisons were performed using one-way ANOVA analysis, with Dunnett’s multiple test, where *p* < 0.05 was considered statistically significant. 

## 4. Conclusions

Research into the production and analysis of ascorbic acid derivatives has provided crucial understanding of their potential use in skin treatment and care. This investigation focused on creating a new type of persulfated ascorbic acid glycoside (AAGS). This novel persulfated ascorbic acid glycoside derivative (AAGS) was synthesized, its safety profile and bioactivities were evaluated, and preformulation studies were conducted to assess its stability and interactions with excipients. Throughout these investigations, comparisons were made with its parent ingredients to evaluate its potential as a prospective cosmetic ingredient. Its noteworthy safety profile, characterized by non-sensitizing characteristics, could constitute a point in favor of further investigation in skincare formulations, together with the anti-allergic potential by impeding the maturation of THP-1 monocyte cells triggered by the potent allergen DNFB. AAGS demonstrated a degree of DPPH radical scavenging activity (EC_50_ = 5.28 ± 0.36 µM) comparable to that of its parent compound, AA, suggesting that the molecular modification did not significantly alter its antioxidant activity. Preformulation studies emphasized AAGS stability across a spectrum of pH solutions close to the skin pH, offering an advantage over AA. Although its stability in the presence of metal solutions was similar to that of AA, AAGS demonstrated superior photostability, a crucial attribute for photoprotective cosmetic formulations. Compatibility studies conducted on AA, AAG, and AAGS with various commonly used topical excipients indicated the favorable compatibility of AAGS with HEC, along with a compatibility profile similar to that of AA and AAG when tested with the other excipients. AAGS holds promise as a potential substitute for AA derivatives in specialized skincare solutions, namely in the development of skincare products aimed at addressing specific dermatological concerns, such as sensitive or allergy-prone skin, or in anti-aging and sunscreen formulations, suggesting a potential to fight oxidative stress and protect the skin from UV-induced photodamage. Overall, the findings suggest that AAGS could serve as a novel ingredient in skincare formulations, particularly in situations where stability and safety properties are paramount, or where AA or other derivatives are not optimal choices.

## Figures and Tables

**Table 1:** Results of the compatibility study between AA, AAG, and AAGS and the excipients.

	Thermoanalytical Data	Stearilic Alcohol (SA)	Carbopol 980 (CP)	Ethylenediamine Tetraacetic Acid (EDTA)	Hydroxyethylcellulose (HEC)	Miglyol 812 (MIG)	Potassium Cetyl Phosphate (PCP)	Tocopherol (TP)
**AA**	T_onset_ (°C) = 192.9 T_peak_ (°C) = 197.1 ΔH (J/g) = −328.6							
**AAG**	T_onset_ (°C) = 173.0 T_peak_ (°C) = 176.4 ΔH (J/g) = −139.8							
**AAGS**	T_onset_ (°C) = 195.0 T_peak_ (°C) = 200.5 ΔH (J/g) = 223.1							

**Green:** no interference in the thermograms, peaks well-defined; **yellow:** weak interference, decrease of intensity of at least one peak; **orange:** medium interference with evident decrease and/or appear/disappear of peaks; **red:** strong interference, alterations of all the peaks of the compounds.

**Table 2:** Summary table of the results of all the tests carried out for AA, AAG, and AAGS.

Cytotoxicity	HaCaT Cells: AAGS > AA > AAG RAW264.7 and THP-1 Cells: AA, AAG, and AAGS with Similar Profiles
**Anti-inflammatory activity**	AA > AAG > AAGS
**Non-sensitization profile**	AA, AAG, and AAGS with non-sensitizer profile
**Anti-allergic activity**	AA > AAGS > AAG
**Mitochondrial antioxidant activity**	AA, AAG, and AAGS with no pro-oxidant profile % inhibition of rotenone-induced SO_2_^−^ production: AA > AAG > AAGS
**DPPH activity**	AA > AAGS > AAG
**pH stability**	AAGS > AAG > AA
**Thermostability**	AAGS > AAG > AA
**Metals stability**	AAG > AAGS ≈ AA
**Photostability**	AAGS > AAG > AA
**Excipient compatibility**	AA, AAG and AAGS with similar profile

## Data Availability

Data are contained within the article and Supplementary Materials.

## Supplementary Materials

The following supporting information can be downloaded at: https://www.mdpi.com/article/10.3390/molecules29194604/s1, Figure S1: Obtained chromatogram for AAGS by HPLC-DAD analysis (column C18; mobile phase: 25 mM of tetrabutylammonium and acetonitrile (38:62)). Figure S2: IR (KBr) spectra: dark blue = 2-*O*-α-d-glucopyranosyl-l-ascorbic acid persulfate (AAGS), light blue = ascorbic acid 2-glucoside, and red = ascorbic acid. Figure S3: ^1^H NMR spectra of ascorbic acid 2-glucoside (AAG) and 2-*O*-α-d-glucopyranosyl-l-ascorbic acid persulfate (AAGS). * Measured in DMSO-*d*_6_ at 300.13 MHz. Figure S4: ^13^C NMR spectrum of 2-*O*-α-d-glucopyranosyl-l-ascorbic acid persulfate (AAGS). * Measured in DMSO-*d*_6_ at 75.47 MHz. Figure S5: ESI-HRMS of 2-*O*-α-d-glucopyranosyl-l-ascorbic acid persulfate (AAGS). Figure S6: Stability studies at different pH for compounds AA; AAG and AAGS. (a) pH 2, (b) pH 3, (c) pH 4, (d) pH 7, and (e) pH 8. Results are expressed as % of absorbance normalized to the absorbance at the time t = 0, in triplicate. Figure S7: Stability studies for compounds AA, AAG, and AAGS at pH 5, at (a) 4 °C, and (b) 40 °C. Results are expressed as % of absorbance normalized to the absorbance at the time t = 0, in triplicate. Figure S8: Stability studies for compounds AA, AAG, and AAGS at pH 5 for different metals, (a) Fe(III), (b) Cu(II), and (c) Ca(II). Results are expressed as % of absorbance normalized to the absorbance at the time t = 0, in triplicate.; Figure S9: Thermograms of individual compounds and the respective equivalent (1:1) mixtures for the compounds (a) AA, (b) AAG, (c) AAGS, (d) AA + stearic acid, (e) AAG + stearic acid, (f) AAGS + stearic acid, (g) AA + carbopol, (h) AAG + carbopol, (i) AAGS + carbopol, (j) AA + EDTA, (k) AAG + EDTA, (l) AAGS + EDTA, (m) AA + hydroxyethylcellulose, (n) AAG + hydroxyethylcellulose, (o) AAGS + hydroxyethylcellulose, (p) AA + miglyol, (q) AAG + miglyol, (r) AAGS + miglyol, (s) AA + potassium cetyl phosphate, (t) AAG + potassium cetyl phosphate, (u) AAGS + potassium cetyl phosphate, (v) AA + tocopherol (w) AAG + tocopherol, (x) AAGS + tocopherol. Red line: antioxidant (AA, AAG or AAGS), blue line (excipients), and green line: 1:1 (*w*/*w*) mixtures. Table S1: ^1^H and ^13^C NMR data of 2-*O*-α-d-glucopyranosyl-l-ascorbic acid persulfate (AAGS) and ascorbic acid persulfate (AscS) (DMSO-*d*_6_). Table S2: DPPH scavenging activity of AA, AAG and AAGS given by EC_50_ values (µM). References [39,40] are cited in the Supplementary Materials.
