# Supplementary material for: Persulfated Ascorbic Acid Glycoside as a Safe and Stable Derivative of Ascorbic Acid for Skin Care Application
Source: Molecules. 2024 Sep 27;29(19):4604. doi: 10.3390/molecules29194604 (PMC11477482; doi:10.3390/molecules29194604)
Supplement: Supplementary file 1 [file molecules-29-04604-s001.zip › molecules-3112043-supplementary.pdf]

molecules

Supplementary Materials

Persulfated Ascorbic Acid Glycoside as a Safe and Stable Derivative of Ascorbic Acid for Skin Care Application

Ana Jesus [1,2,†], Marta Correia-da-Silva [3,4,†], Catarina Confraria [3], Sílvia Silva [5], Gonçalo Brites [6,7], Ana I. Sebastião [6,7], Mylène Carrascal [7,8], Madalena Pinto [3,4], Honorina Cidade [3,4,9], Paulo Costa [1,2], Maria T. Cruz [6,7,*], Emília Sousa [3,4,*] and Isabel F. Almeida [1,2]

[1] Associate Laboratory i4HB—Institute for Health and Bioeconomy, University of Porto, 4050-313 Porto, Portugal; anaaimjesus@gmail.com (A.J.); pccosta@ff.up.pt (P.C.); ifalmeida@ff.up.pt (I.F.A.)
[2] UCIBIO—Applied Molecular Biosciences Unit, Laboratory of Pharmaceutical Technology, Faculty of Pharmacy, University of Porto, 4050-313 Porto, Portugal
[3] Laboratory of Organic and Pharmaceutical Chemistry, Department of Chemical Sciences, Faculty of Pharmacy, University of Porto, 4050-313 Porto, Portugal; m_correiadasilva@ff.up.pt (M.C.-d.-S.); catarinailc@gmail.com (C.C.); madalena@ff.up.pt (M.P.); hcidade@ff.up.pt (H.C.)
[4] CIIMAR—Interdisciplinary Center of Marine and Environmental Research, Avenida General Norton de Matos S/N, 4450-208 Matosinhos, Portugal
[5] Laboratory of Pharmaceutical Technology, Department of Drug Sciences, Faculty of Pharmacy, University of Porto, 4050-313 Porto, Portugal; silvarfsilvia@gmail.com
[6] Faculty of Pharmacy, University of Coimbra, 3004-531 Coimbra, Portugal; g.sousabrites3@gmail.com (G.B.); anaisabelsebastiao@gmail.com (A.I.S.)
[7] CNC—Center for Neurosciences and Cell Biology, 3004-504 Coimbra, Portugal; mylenecarrascal87@gmail.com
[8] Labor Qualitas, Tecnimede Group, Rua da Tapada Grande, Abrunheira, 2710-089 Sintra, Portugal
[9] UNIPRO—Oral Pathology and Rehabilitation Research Unit, University Institute of Health Sciences (CESPU), 4585-116 Gandra, Portugal
* Correspondence: trosete@ff.uc.pt (M.T.C.); esousa@ff.up.pt (E.S.)
† These authors contributed equally to this work.

molecules MDPI

Table S1 ^{1}H and ^{13}C NMR data of 2-O-*α*-*D*-glucopyranosyl-*L*-ascorbic acid persulfate (AAGS) and ascorbic acid persulfate (AscS) (DMSO-d_6).

$^{1}H^{a}$	δ (ppm)		$^{13}C^{b}$	δ (ppm)	
	AAGS	AscS*		AAGS	AscS*
**1**	-	-	1	176.5	173.1
**2**	-	-	2	114.2	95.7
**3**	-	-	3	173.6	154.2
**4**		4.29 (m)	4	114.2	92.4
**5**	3.94-4.10 (m)	4.52 (q)	5	70.4	70.2
**6a**		4.14 (q)	6	63.3	63.1
**6b**	3.70-3.83 (m)	3.73 (t)			
**1′**	5.53 (d, J = 2.9 Hz)	-	1′	97.3	-
**2′**	4.52-4.62 (m)	-	2′	74.0	-
**3′**	4.52-4.62 (m)	-	3′	75.4	-
**4′**	4.31 (brt)	-	4′	71.0	-
**5′**	4.13-4.18 (m)	-	5′	74.0	-
**6′a**	3.94-4.10 (m)	-	6′	65.9	-
**6′b**	3.70-3.83 (m)	-			

[a]Measured in DMSO-d_6 at 300.13 MHz. Values in parts per million (δ_H).
[b]Measured in DMSO-d_6 at 75.47 MHz. Values in parts per million (δ_C).
*Literature assignments (1)

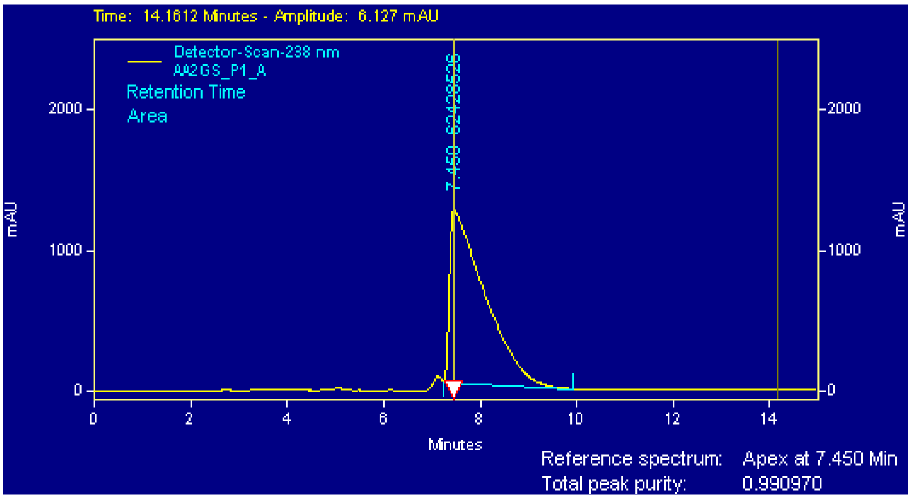

Figure S1 Obtained chromatogram for AAGS by HPLC-DAD analysis (column C18; mobile phase: 25 mM of tetrabutylammonium and acetonitrile (38:62)).

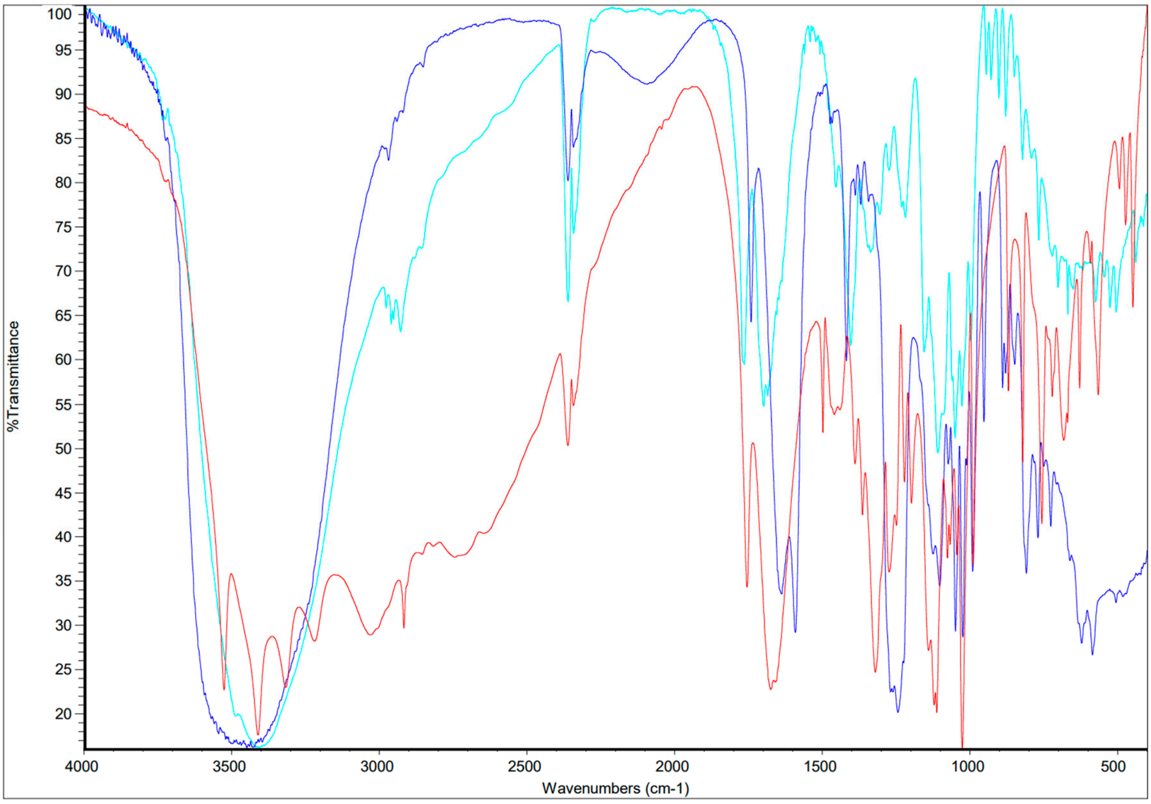

Figure S2 IR (KBr) spectra: dark blue= 2-O-*α*-*D*-glucopyranosyl-*L*-ascorbic acid persulfate (AAGS), light blue= ascorbic acid 2-glucoside, and red= ascorbic acid.

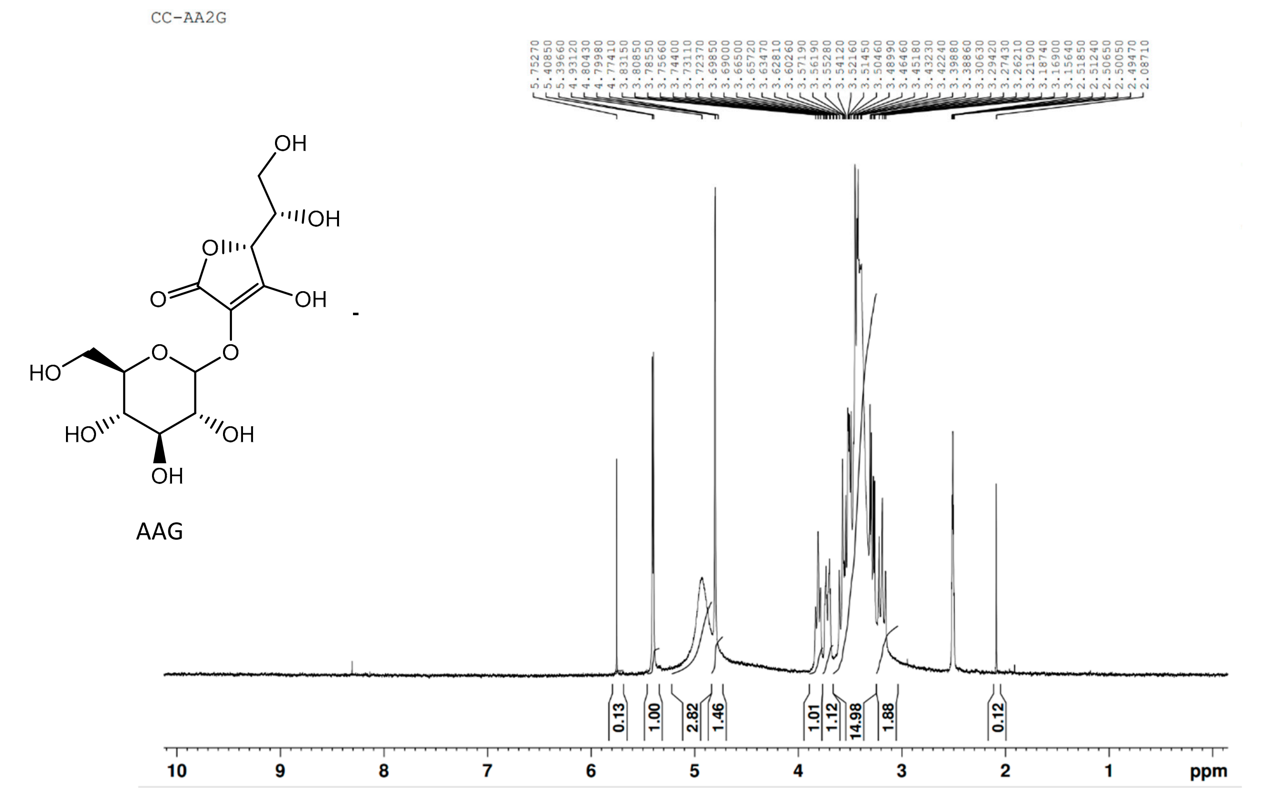

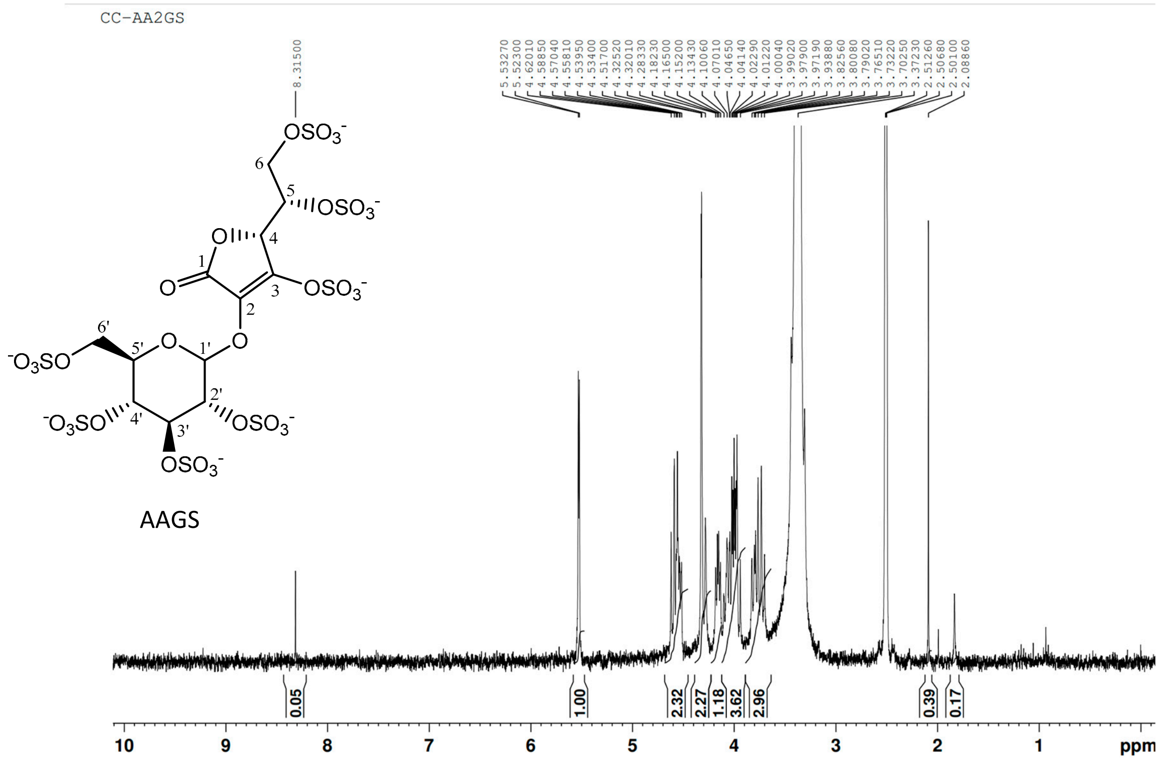

Figure S3 ^{1}H NMR spectra of ascorbic acid 2-glucoside (AAG) and 2-O-*α*-*D*-glucopyranosyl-*L*-ascorbic acid persulfate (AAGS). *Measured in DMSO-d_6 at 300.13 MHz.

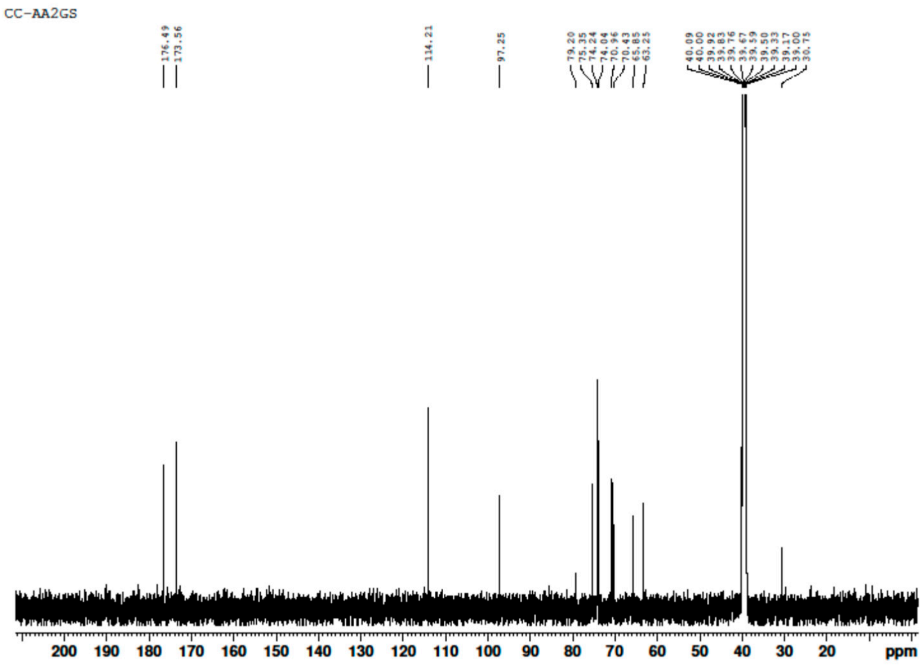

Figure S4 ^{13}C NMR spectrum of 2-O-*α*-*D*-glucopyranosyl-*L*-ascorbic acid persulfate (AAGS). *Measured in DMSO-*d6* at 75.47 MHz.

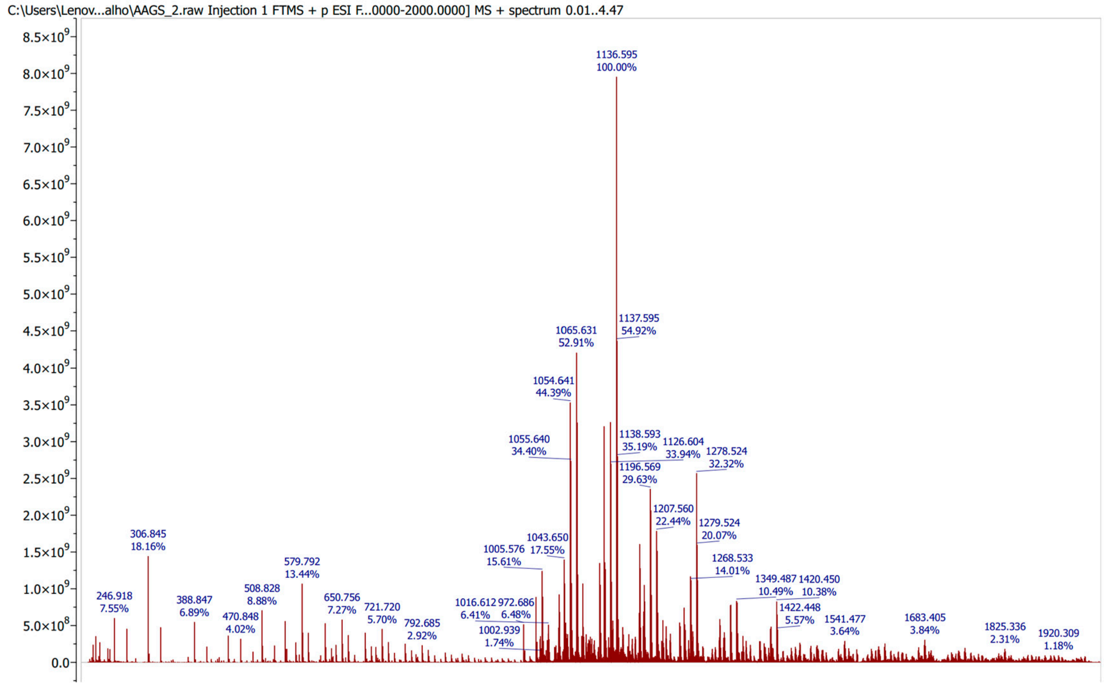

Figure S5. ESI-HRMS of 2-O-*α*-*D*-glucopyranosyl-*L*-ascorbic acid persulfate (AAGS). **Experimental conditions**. Analysis was done on an Orbitrap Exploris 120 mass spectrometer (Thermo Fischer Scientific, Bremen, Germany) controlled by *Orbitrap Exploris Tune Application 2.0.185.35* and *Xcalibur 4.4.16.14*. The capillary voltage of the electrospray ionization source (ESI) was set to 3.4 kV and 2 kV for positive and negative mode. The capillary temperature was 320ºC. The sheath gas and auxiliary gas flow rate were at 5 (arbitrary unit as provided by the software settings). The resolution of MS scan was 60 000. Data dependent MS/MS was performed on HCD using nitrogen as gas with collision energy settings of 30 V. The m/z range were 200-2000 Da. The resolution of SIM MS scan was 60,000. MS data handling software (Xcalibur QualBrowser software, Thermo Fischer Scientific) was used to search for predicted metabolites by their *m*/*z* value and MS/MS value.

Table S2 - DPPH scavenging activity of AA, AAG and AAGS given by EC_{50} values (µM).

Compounds	EC_{50}* ± SD (µM)
AA#	1.54 ± 0.21
AAG	9.15 ± 0.13
AAGS	5.28 ± 0.36

* EC50: Concentration of a compound required to scavenge 50% of the initial DPPH radical.
SD: Standard deviation
EC_{50} of AA in DPPH assay (literature value) = 16.3 ± 0.569 µM (2).

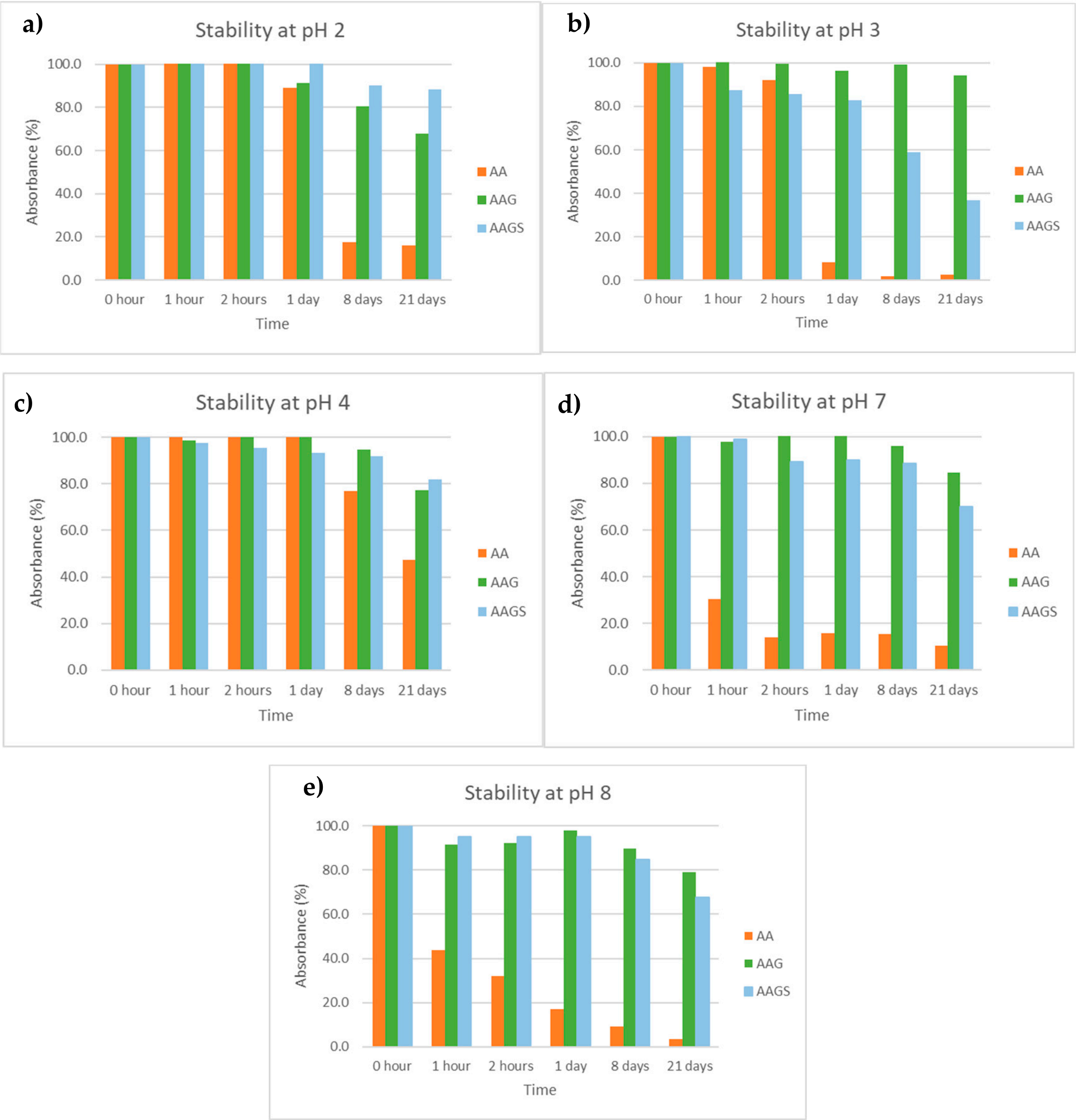

Figure S6 Stability studies at different pH for compounds AA; AAG and AAGS. a) pH 2, b) pH 3, c) pH 4, d) pH 7 and e) pH 8. Results are expressed as % of absorbance normalized to the absorbance at the time t=0, in triplicate.

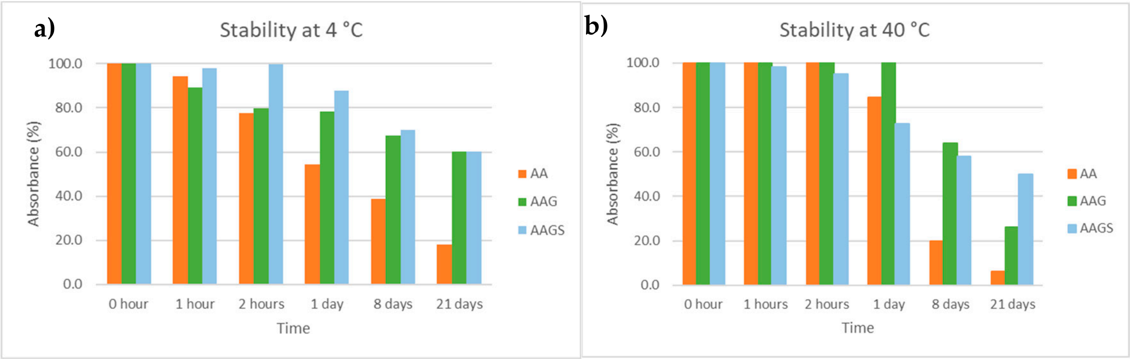

Figure S7 Stability studies for compounds AA; AAG and AAGS at pH 5, at a) 4 ºC, and b) 40 ºC. Results are expressed as % of absorbance normalized to the absorbance at the time t=0, in triplicate.

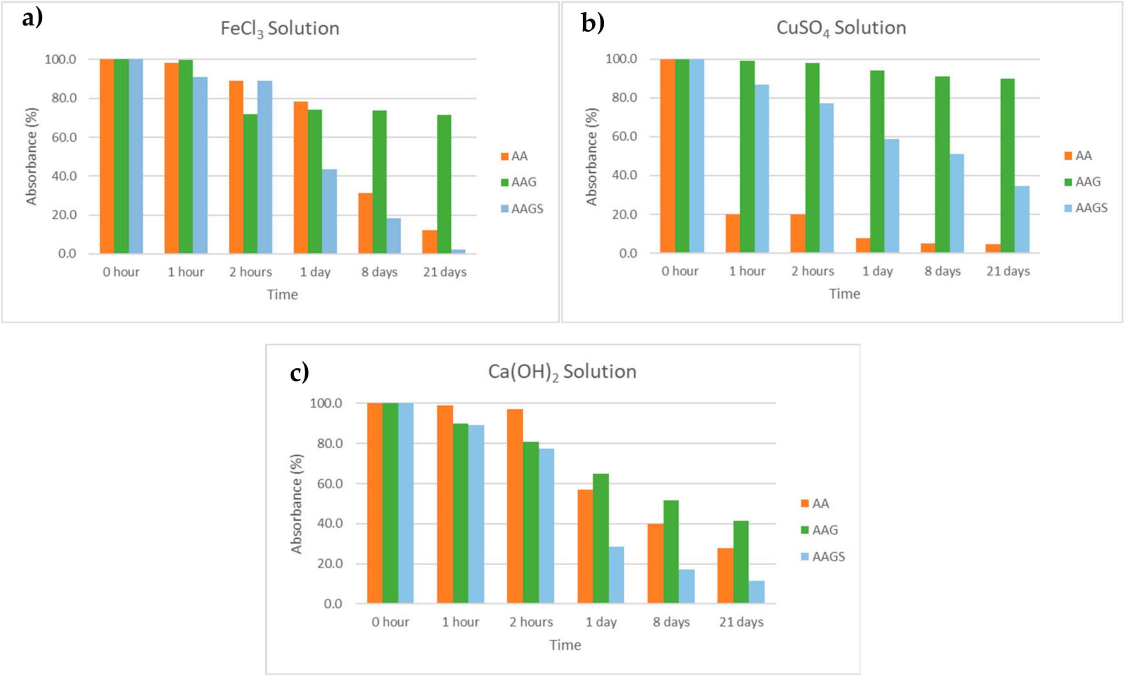

Figure S8 Stability studies for compounds AA; AAG and AAGS at pH 5, for different metals, a) Fe(III), b) Cu(II) and c) Ca(II). Results are expressed as % of absorbance normalized to the absorbance at the time t=0, in triplicate.

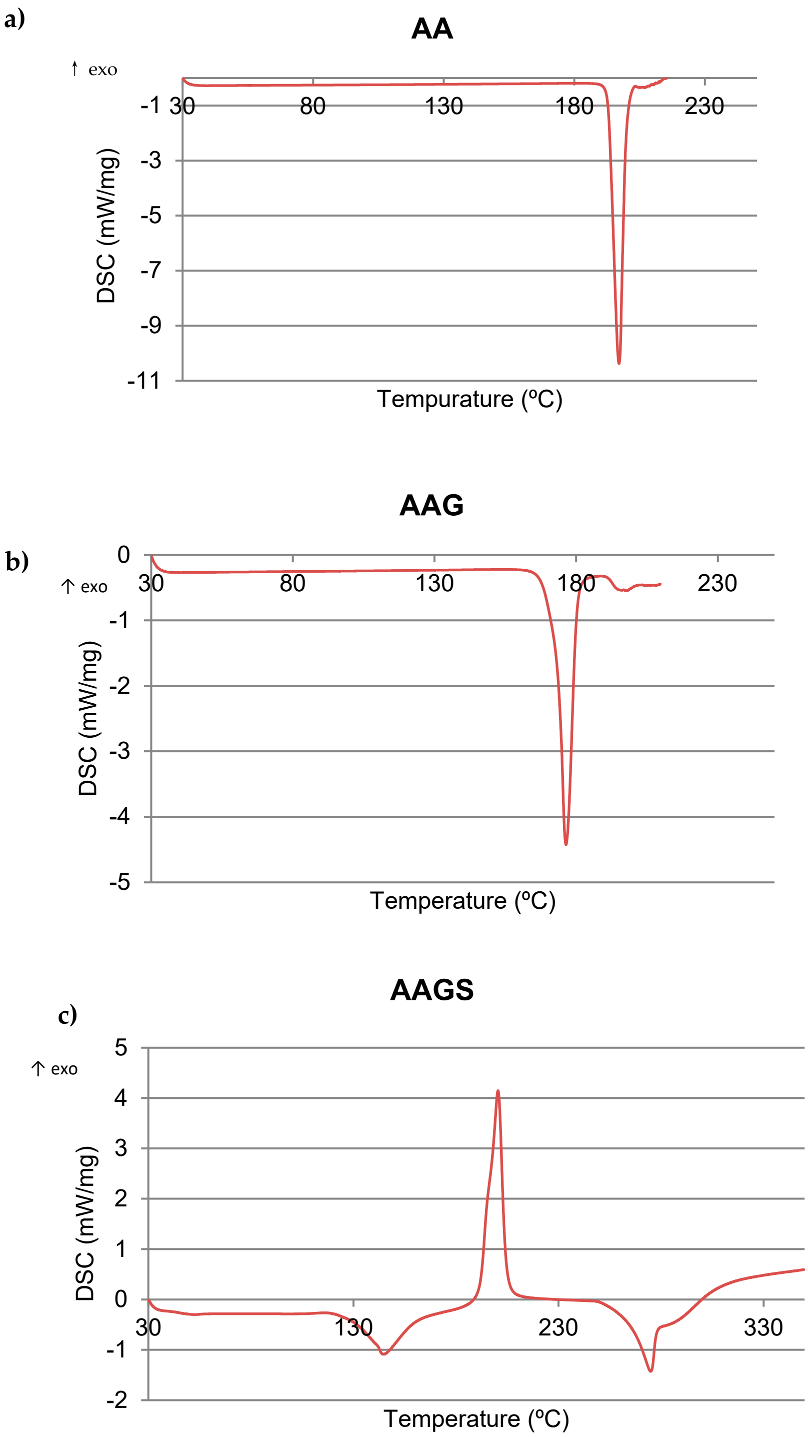
a)
AA
↑ exo
DSC (mW/mg)
-1
-3
-5
-7
-9
-11
30
80
130
180
230
Tempurature (ºC)
b)
AAG
↑ exo
DSC (mW/mg)
0
-1
-2
-3
-4
-5
30
80
130
180
230
Temperature (ºC)
c)
AAGS
↑ exo
DSC (mW/mg)
5
4
3
2
1
0
-1
-2
30
130
230
330
Temperature (ºC)

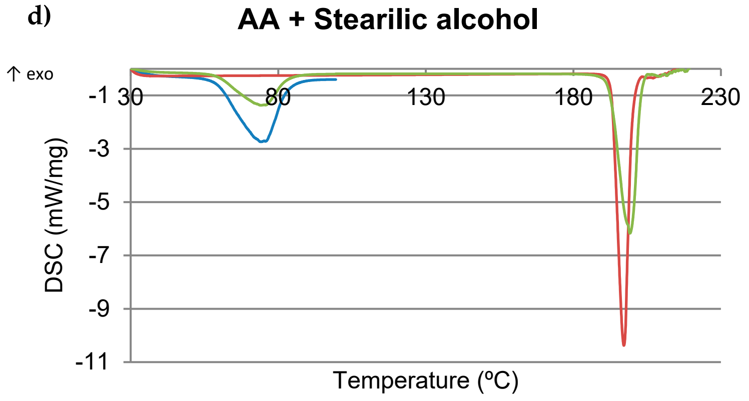
d)
AA + Stearilic alcohol
↑ exo
DSC (mW/mg)
-1
-3
-5
-7
-9
-11
30
80
130
180
230
Temperature (ºC)

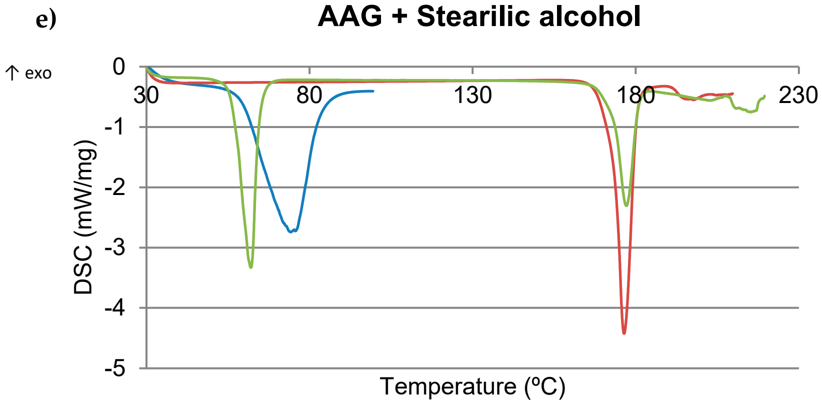
e)
AAG + Stearilic alcohol
↑ exo
DSC (mW/mg)
0
-1
-2
-3
-4
-5
30
80
130
180
230
Temperature (ºC)

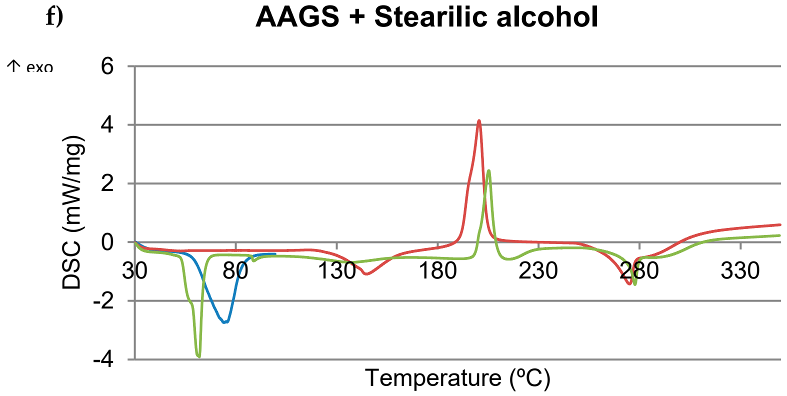
f)
AAGS + Stearilic alcohol
↑ exo
DSC (mW/mg)
6
4
2
0
-2
-4
30
80
130
180
230
280
330
Temperature (ºC)

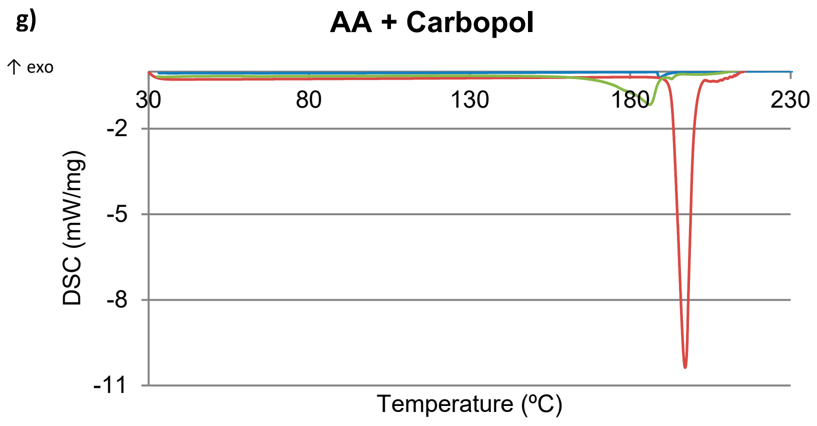
g)
AA + Carbopol
↑ exo
30
80
130
180
230
-2
-5
-8
-11
DSC (mW/mg)
Temperature (ºC)

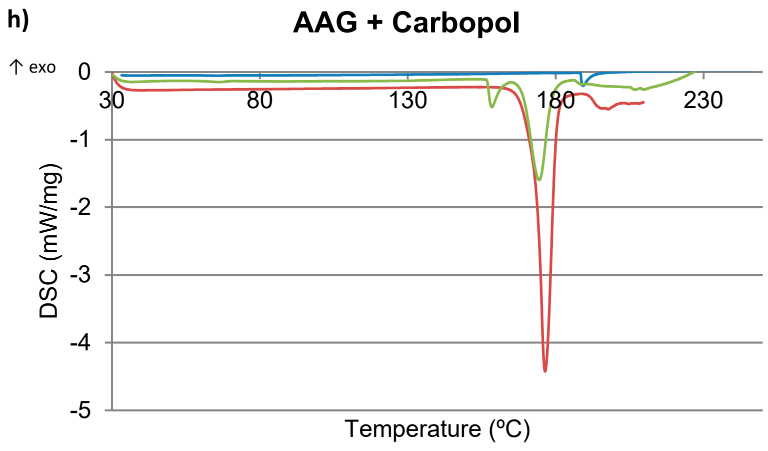
h)
AAG + Carbopol
↑ exo
0
30
80
130
180
230
-1
-2
-3
-4
-5
DSC (mW/mg)
Temperature (ºC)

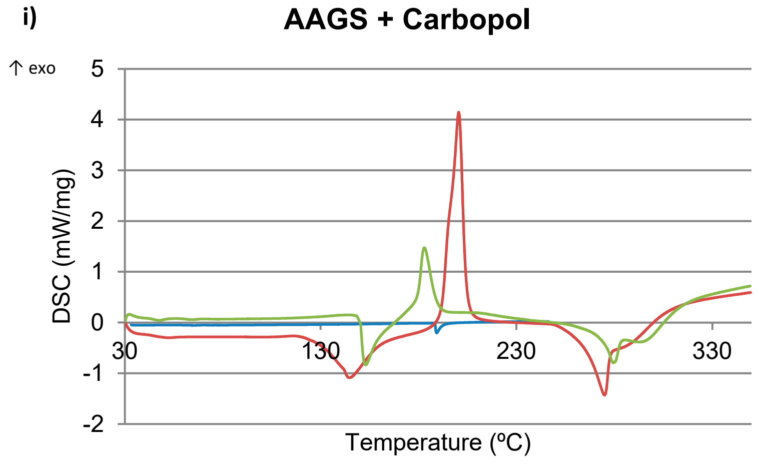
i)
AAGS + Carbopol
↑ exo
5
4
3
2
1
0
-1
-2
30
130
230
330
DSC (mW/mg)
Temperature (ºC)

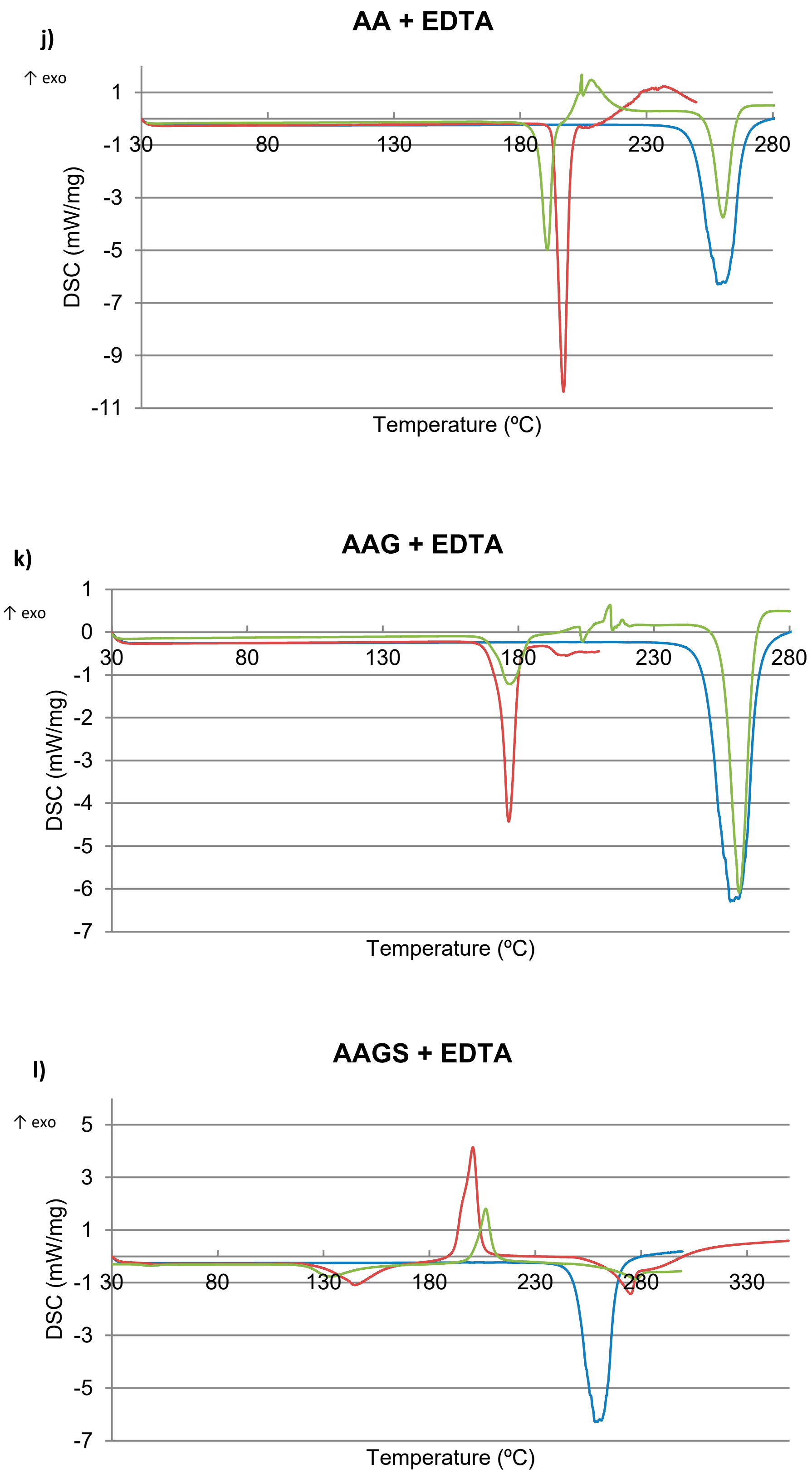
j)
AA + EDTA
↑ exo
1
-1
-3
-5
-7
-9
-11
30
80
130
180
230
280
DSC (mW/mg)
Temperature (ºC)
k)
AAG + EDTA
↑ exo
1
0
-1
-2
-3
-4
-5
-6
-7
30
80
130
180
230
280
DSC (mW/mg)
Temperature (ºC)
l)
AAGS + EDTA
↑ exo
5
3
1
-1
-3
-5
-7
30
80
130
180
230
280
330
DSC (mW/mg)
Temperature (ºC)

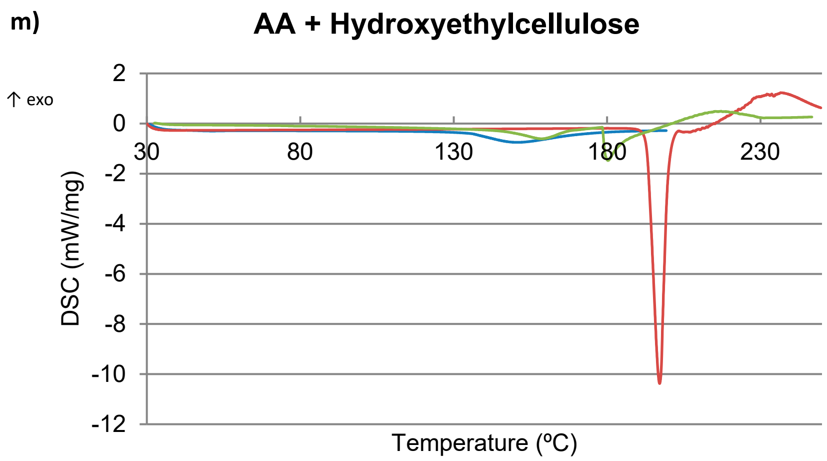
m)
AA + Hydroxyethylcellulose
↑ exo
2
0
-2
-4
-6
-8
-10
-12
30
80
130
180
230
DSC (mW/mg)
Temperature (ºC)

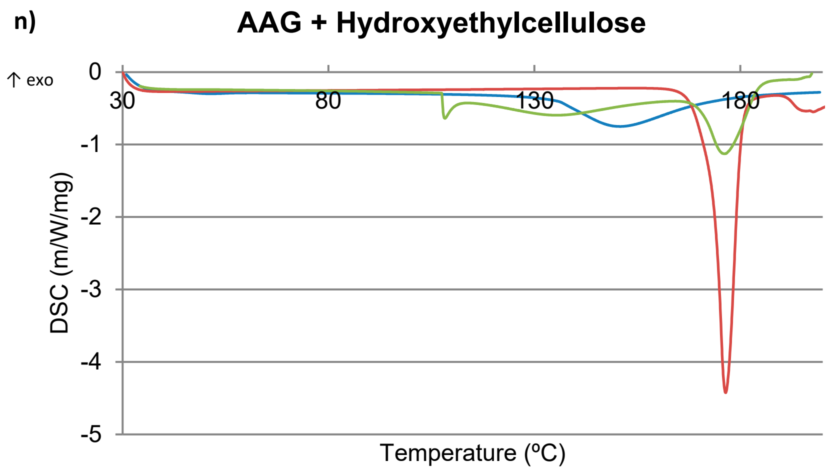
n)
AAG + Hydroxyethylcellulose
↑ exo
0
-1
-2
-3
-4
-5
30
80
130
180
DSC (m/W/mg)
Temperature (ºC)

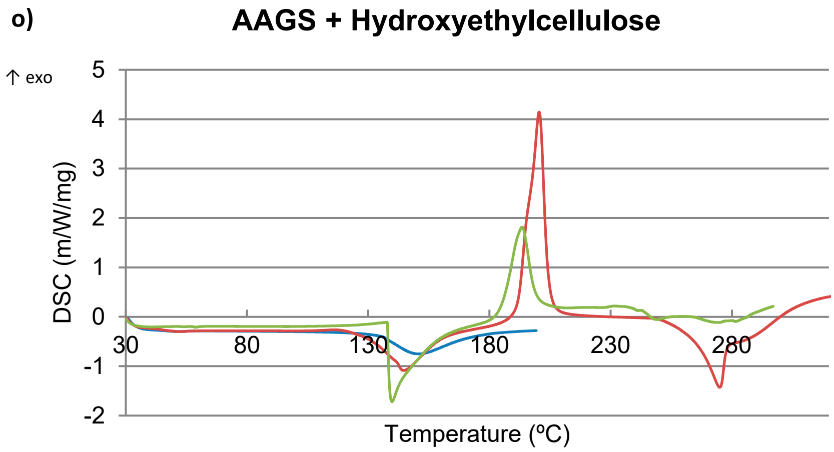
o)
AAGS + Hydroxyethylcellulose
↑ exo
5
4
3
2
1
0
-1
-2
30
80
130
180
230
280
DSC (m/W/mg)
Temperature (ºC)

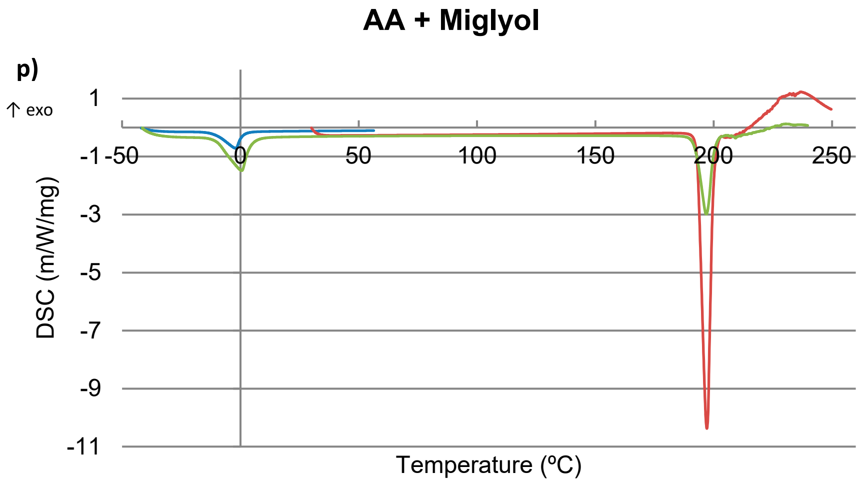
AA + Miglyol
p)
↑ exo
1
-1
-3
-5
-7
-9
-11
-50
0
50
100
150
200
250
DSC (m/W/mg)
Temperature (ºC)

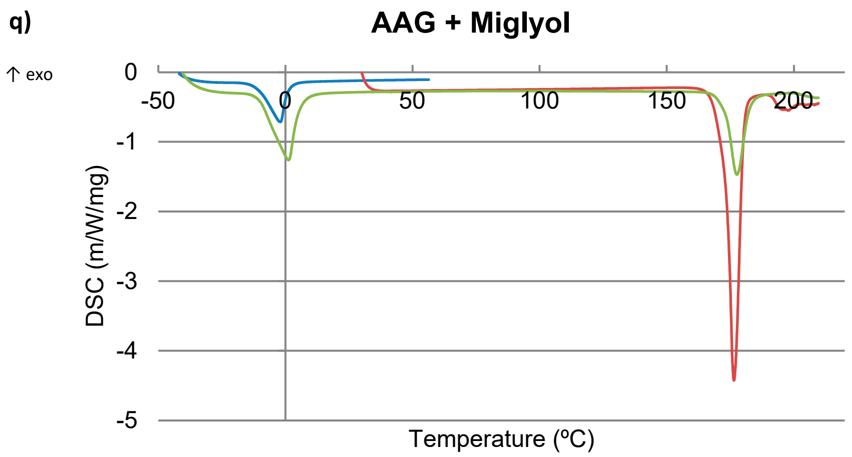
q)
AAG + Miglyol
↑ exo
0
-1
-2
-3
-4
-5
-50
0
50
100
150
200
DSC (m/W/mg)
Temperature (ºC)

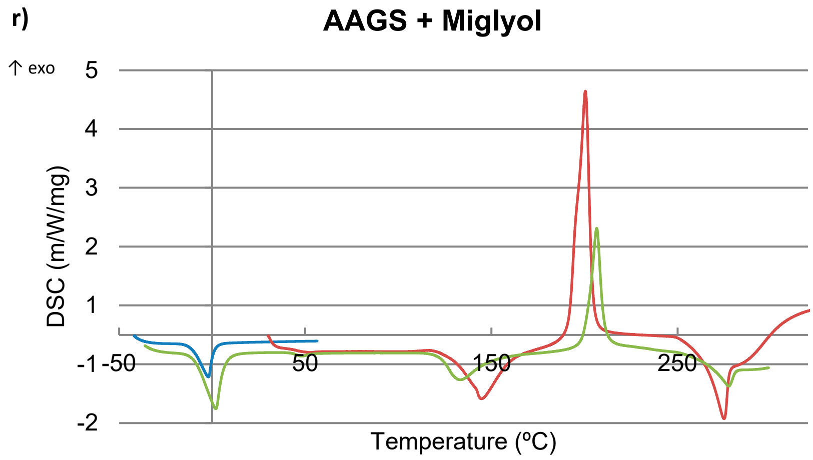
r)
AAGS + Miglyol
↑ exo
5
4
3
2
1
-1
-2
-50
50
150
250
DSC (m/W/mg)
Temperature (ºC)

s)

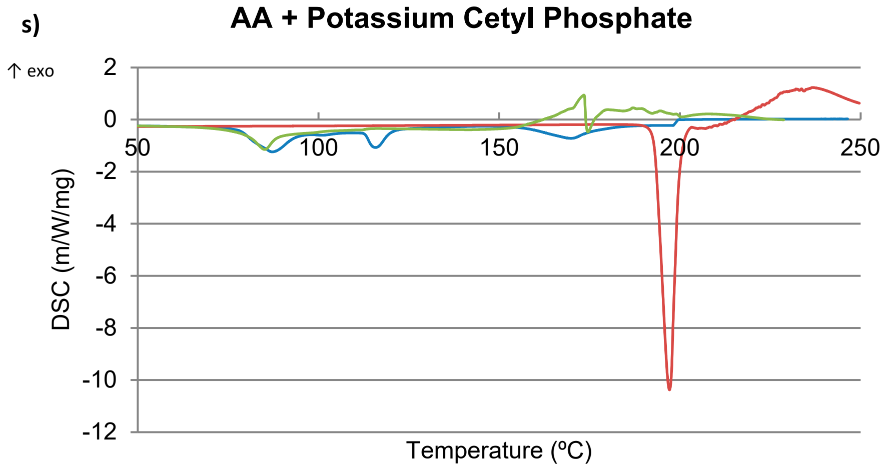
AA + Potassium Cetyl Phosphate
↑ exo
DSC (m/W/mg)
Temperature (ºC)
2
0
-2
-4
-6
-8
-10
-12
50
100
150
200
250

t)

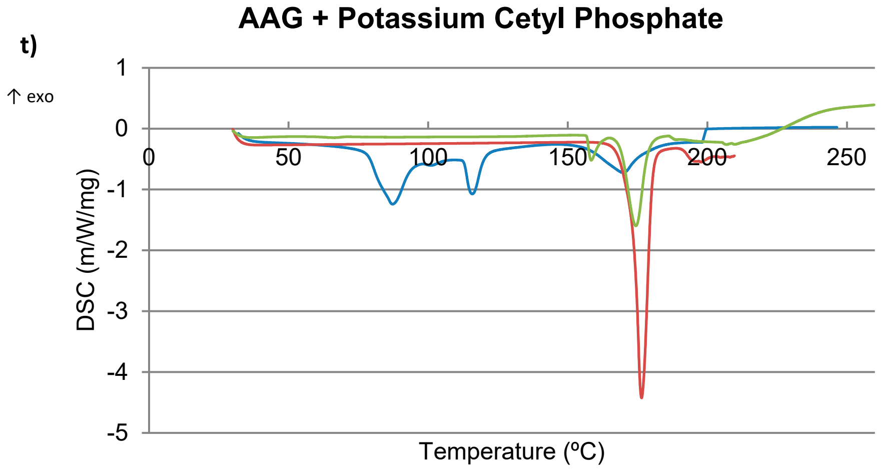
AAG + Potassium Cetyl Phosphate
↑ exo
DSC (m/W/mg)
Temperature (ºC)
1
0
-1
-2
-3
-4
-5
0
50
100
150
200
250

u)

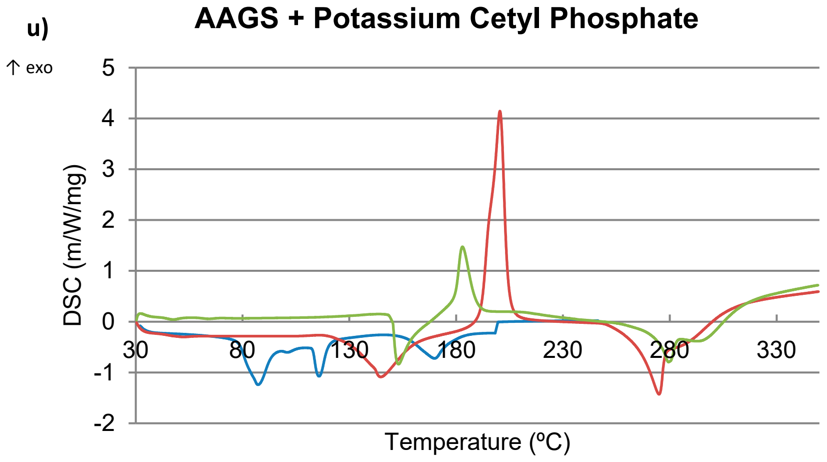
AAGS + Potassium Cetyl Phosphate
↑ exo
DSC (m/W/mg)
Temperature (ºC)
5
4
3
2
1
0
-1
-2
30
80
130
180
230
280
330

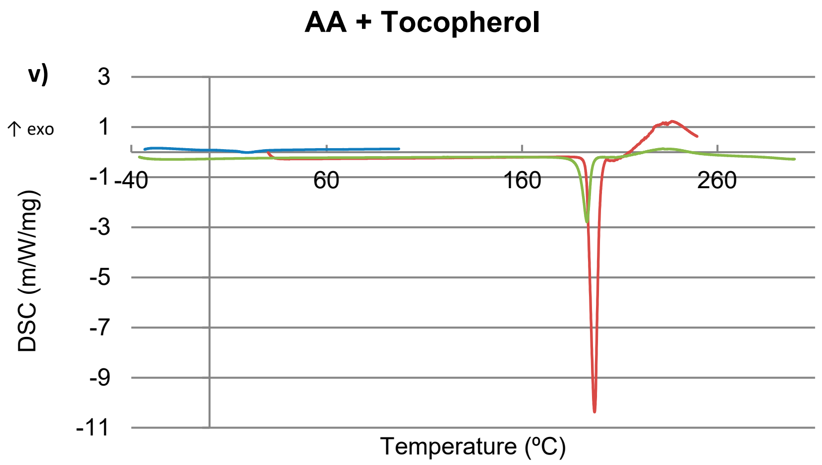

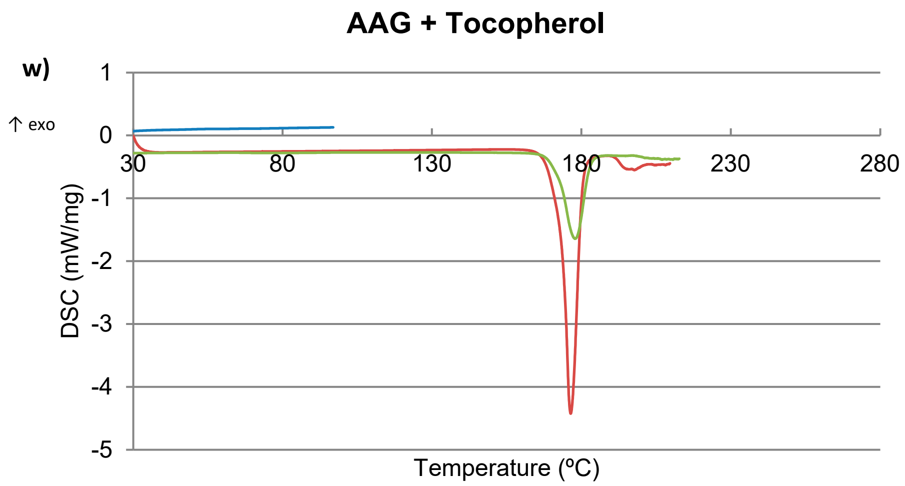

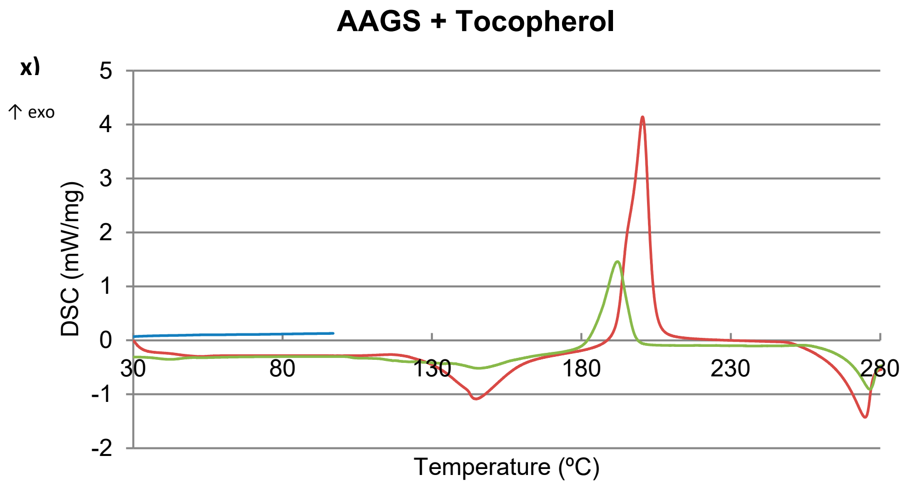

Figure S9 Thermograms of individual compounds and the respective equivalent (1:1) mixtures for the compounds a) AA, b) AAG, c) AAGS, d) AA + stearilic acid, e) AAG + stearilic acid, f) AAGS + stearilic acid, g) AA + carbopol, h) AAG + carbopol, i) AAGS + carbopol, j) AA + EDTA, k) AAG + EDTA, l) AAGS + EDTA, m) AA + hydroxyethylcellulose, n) AAG + hydroxyethylcellulose, o) AAGS + hydroxyethylcellulose, p) AA + miglyol, q) AAG + miglyol, r) AAGS + miglyol, s) AA + potassium cetyl phosphate, t) AAG + potassium cetyl phosphate, u) AAGS + potassium cetyl phosphate, v) AA + tocopherol w) AAG + tocopherol, x) AAGS + tocopherol. Red line: antioxidant (AA, AAG or AAGS), blue line (excipients), and green line: 1:1 (w/w) mixtures.

References:

1. Correia-da-Silva M, Sousa E, Duarte B, Marques F, Cunha-Ribeiro LM, Pinto MM. Dual anticoagulant/antiplatelet persulfated small molecules. Eur J Med Chem. 2011;46(6):2347-58.
2. Afsar T, Razak S, Shabbir M, Khan MR. Antioxidant activity of polyphenolic compounds isolated from ethyl-acetate fraction of Acacia hydaspica R. Parker. (1752-153X (Print)).
